# Vitamin D: What We Know and What We Still Do Not Know About Vitamin D in Preterm Infants—A Literature Review

**DOI:** 10.3390/children12030392

**Published:** 2025-03-20

**Authors:** Ioana Andrada Radu, Maria Livia Ognean, Laura Ștef, Doina Ileana Giurgiu, Manuela Cucerea, Cristian Gheonea

**Affiliations:** 1 Doctoral School, University of Medicine and Pharmacy, 200349 Craiova, Romania; ioana.radu@ulbsibiu.ro; 2 Faculty of Medicine, Lucian Blaga University, 550169 Sibiu, Romania; 3 Clinical County Emergency Hospital, 550245 Sibiu, Romania; 4 Department of Neonatology, George Emil Palade University of Medicine, Pharmacy, Science and Technology of Targu Mures, 540142 Targu Mures, Romania; manuela.cucerea@umfst.ro; 5 Department of Pediatrics, University of Medicine and Pharmacy, 200349 Craiova, Romania

**Keywords:** preterm infants, vitamin D, 25(OH)D levels, vitamin D deficiency, enteral supplementation

## Abstract

Background/Objectives: Preterm infants represent a population group at increased risk for vitamin D deficiency (VDD) and for its negative impact on various outcomes like metabolic bone disease or rickets, respiratory complications like respiratory distress syndrome and the development of bronchopulmonary dysplasia, necrotizing enterocolitis, or retinopathy of prematurity. Methods: Despite the growing interest in vitamin D research, there is still uncertainty regarding clear recommendations for each high-risk category of premature infants concerning the optimal dosage, optimal product, and timing for initiating vitamin D supplementation to prevent VDD. Results: An analysis of the literature suggests that early intervention for the optimal enteral supplementation of vitamin D is not only successful in achieving higher 25-hydroxi-vitamin D (25(OH)D) at one month but is also linked with improved outcomes. Conclusions: The traditional concepts and current recommendations for assessing vitamin D status and optimal supplementation need to be revised. Since parenteral nutrition, fortified mothers’ own milk, and special formula for preterm infants cannot provide adequate vitamin D levels, initiating oral supplementation soon after birth is essential to correct VDD in preterm infants.

## 1. Introduction

In the last decade, an increased concern has arisen worldwide regarding vitamin D deficiency (VDD) among highly vulnerable groups of the population, like pregnant and lactating mothers and their infants, especially preterm infants. Various risk factors and influences have been evaluated in research studies, but the evidence indicates that a one-size-fits-all dose regimen for vitamin D supplementation is not appropriate for all population groups globally [1,2,3]. Therefore, the optimal 25(OH)D levels remain a subject of debate among experts, while VDD seems to be a global health problem.

It was estimated that more than a billion people worldwide have deficient or insufficient vitamin D status, as defined by the Institute of Medicine (IOM) [4] (Table 1). The risk may be even higher in Europe, as most foods are not fortified with vitamin D [3,4,5].

One of the population groups at high risk for vitamin D deficiency (VDD) and for its negative impact on various outcomes is the vulnerable group of preterm infants [8,9,10]. Vitamin D insufficiency among pregnant women increases the risk for VDD in newborns, especially in preterm infants, as the risk factors for preterm birth overlap with those for VDD [11,12,13,14,15,16,17,18,19,20,21]. Preterm infants depend on maternal vitamin D levels in utero and vitamin D in their dietary intake after birth [18,22]. Fetuses cannot produce vitamin D and most of their vitamin D is transferred across the placenta during the third trimester of pregnancy. As expected, a higher prevalence of VDD was found in preterm infants when compared to term newborns [8,23,24]. Multiple studies reported an association between low vitamin D status at birth and lower gestational ages, as well as increased rates of VDD with advancing gestational age (GA) [19,25,26]. The prevalence of severe maternal VDD (defined as vitamin D levels below 10 mg/mL) varies significantly, up to 56% [20].

In a recent meta-analysis including 72 studies, maternal vitamin D levels were inversely associated with the risk of preterm birth (risk ratio [RR] 0.67; 95% confidence interval [CI] 0.57–0.79); the dose–response analysis indicated that for each increase of 25 nmol/L in 25(OH)D level, the preterm delivery risk decreased by 6% (RR: 0.94; 95% CI 0.90–0.98) [27]. In 2020, in a review, Kiely et al. [28] showed that there are still uncertainties regarding nutritional needs and the benefits of vitamin D supplementation during pregnancy. Conversely, the multicenter study DALI revealed that vitamin levels ≥ 50 nmol/L were achieved in 97% by 24–28 weeks of gestation and in 98% by 35–37 weeks of gestation among pregnant women receiving vitamin D supplementation compared to placebo (*p* < 0.001) [29]. Additionally, a meta-analysis comprising 42 studies published by Liu et al. [30] and including 11,082 participants demonstrated that vitamin D supplementation with over 400 IU/day as compared to less than 400 IU/day reduced the risk of vitamin D insufficiency (RR 0.51; 95% CI 0.38–0.67) and led to increased neonatal 25(OH)D levels (mean difference 27.72 nmol/L; 95% CI 20.51–34.92). These findings are consistent with those reported in a previous meta-analysis published by Gallo et al. [31], which concluded that vitamin D supplementation during pregnancy significantly increased both maternal and neonatal 25(OH)D levels compared to non-supplemented cases; the authors also noted a significantly increased birth weight (BW) (*p* = 0.012) in infants born to mothers receiving vitamin D supplementation.

Still, there are no clear recommendations for the routine screening of serum concentration of 25(OH)D at birth. Both the World Health Organization (WHO) [32] and the recent Endocrine Society Clinical Practice Guideline [33] recommend against routine screening for vitamin D status or deficiency during pregnancy, but they differ in their recommendations for vitamin D supplementation. The WHO suggests administering 200 IU of vitamin D if VDD is suspected, particularly in cases of limited exposure to sunlight [32]. In contrast, the Endocrine Society advocates for empiric vitamin D supplementation in pregnant women (defining empiric as doses higher than the daily recommended intake established by the IOM and without measuring vitamin D status) as, according to evidence, it lowers the risk of preeclampsia, intrauterine and neonatal death, preterm delivery, and delivery of small-for-gestational-age infants). A dose of 600–5000 IU per day of vitamin D (2500 IU/day in weighted average) in the form of fortified foods and prenatal vitamin formulations containing vitamin D and/or vitamin D supplements is recommended by the Endocrine Society [33].

The publication of the recently updated recommendations from the WHO [32] and the Endocrine Society [33] reignited the discussions around the optimal vitamin D level during pregnancy. In 2020, Tous et al. [34] showed that 25(OH)D levels < 20 ng/mL are associated with increased risk for unfavorable pregnancy outcomes (preeclampsia, pregnancy-induced hypertension, eclampsia, HELLP syndrome). Kurmangali et al. [35] recently suggested that vitamin D concentrations of >30 ng/mL would be necessary to improve pregnancy outcomes and levels of >40 ng/mL would bring even more benefits. This hypothesis is also supported by Grant et al. [36] in a recent review based on an analysis of multiple observational studies, who noted that vitamin D supplementation has more pronounced beneficial effects in pregnant women with the lowest 25(OH)D levels (<10 ng/mL). Criticizing the focus of the current guidelines on bone health, Grant et al. [36] suggests a vitamin D supplementation of 2000–4000 IU/day during pregnancy. This suggestion is in line with the recommendation of Kurmangali et al. [35] that all pregnant women should be screened for vitamin D status and should receive ≥2000 IU vitamin D per day in an individualized manner, according to their 25(OH)D level and risk factors, to ensure their unique physiological needs are met.

The role of vitamin D in the intestinal absorption of calcium and phosphorus and bone mineralization has been well understood for many years [3,33,37,38,39]. Recently, studies revealed a more various range of the effects of vitamin D and its involvement in development, cell growth and differentiation, lung maturation, and development [9,40,41,42]. Furthermore, it plays a critical role in normal fetal brain development and the prevention of brain injury [43,44,45], as well as in modulating the immune system and inflammatory responses [44,46,47,48,49,50]. Possible associations between inadequate levels of vitamin D and the development of bronchopulmonary dysplasia (BPD) [40,51], necrotizing enterocolitis (NEC), or retinopathy of prematurity (ROP) in premature infants were suggested. Vitamin D (calciferol) is not only a fat-soluble vitamin with essential roles in various physiological processes in the body [9] but is also classified as a secosteroid [30,52]; more precisely, according to Yang et al. [18], it functions as a neuroendocrine immune-regulating hormone.

## 2. Vitamin D and Bone Health

Calcium, phosphorus, and vitamin D are the most important nutrients affecting bone formation before and after birth. Beginning in the first trimester of pregnancy, the conversion of 25(OH) D to 1,25(OH)_2_D doubles, resulting in three-fold increase in the active form of vitamin D. This change is accompanied by an increase in the binding protein for vitamin D, which enhances intestinal calcium absorption and supports the mineralization of the fetal skeleton [2,53,54].

Maternal vitamin D supplementation during pregnancy is linked to improved bone mineral content and density, effects that persist into early childhood [55,56,57]. Additionally, it is associated with a decreased incidence of fractures [57,58,59].

However, several factors complicate our understanding of the optimal vitamin D dosage for promoting bone health in preterm infants. These factors include the non-linear relationship between calcium metabolism and bone mineralization, the intricacies and complexity of the vitamin D metabolism, and the existence of a potential threshold above which increasing vitamin D supplementation does not further improve bone accretion. As a result, there is still limited evidence regarding the ideal vitamin D dosage for bone health in this population [60].

In preterm neonates, metabolic bone disease, osteopenia, and rickets are mainly linked to insufficient calcium and phosphorus retention [23,61,62,63]. According to Holick et al. [3], rickets is almost entirely a consequence of insufficient calcium and phosphorus instead of VDD. In preterm infants, inadequate mineral retention may occur secondary to a low mineral intake due to inadequate milk fortification or limited fluid intake in an attempt to avoid fluid overload [63,64]. Intestinal immaturity, feeding difficulties, altered microbioma, and multiple co-morbidities associated with prematurity are increasing the risk of VDD in preterm infants [65,66]. A recent study has reported that the use of fresh pasteurized breast milk is associated with a 5.36-fold increased risk of osteopenia in very-low-birth-weight (VLBW) infants [66]. The findings confirm the prior observation that pasteurization increases the acidity of the milk, which in turn reduces the bioavailability of calcium and phosphorus [67].

Ongoing discussions exist concerning the classification of risk categories, the optimal timing for screening, and the identification of the most relevant biomarkers for metabolic bone disease in preterm infants [68,69,70,71]. More evidence is needed to establish if preterm infants at high risk for rickets or hepatic or renal insufficiency may require routine monitoring and a higher vitamin D dosage for proper nutritional management. A Cochrane protocol regarding vitamin D supplementation to prevent VDD among preterm and low-birth-weight (LBW) infants was published in 2015; the protocol defines VDD by clinical features (rickets, osteopenia, or bone fractures), a serum vitamin D status of <20 ng/mL, or both after using a cumulative dose of ≥200 IU vitamin D for supplementation [72]. The results of the proposed review have yet to be published and, to our knowledge, no other statement has been made concerning this category of premature infants.

## 3. Vitamin D and Respiratory Effects and Outcomes

Several studies focused on the extraskeletal effects of vitamin D, including its immunomodulatory, anti-inflammatory, and antiproliferative effects, highlighting the potential link between VDD in premature infants and adverse neonatal outcomes like respiratory distress (RD), respiratory distress syndrome (RDS), BPD, or acute respiratory lower tract infections (ARLI), and emphasizing the possible disruption of lung development in association with inadequate levels of vitamin D [9,33,40,42,65]. Respiratory distress syndrome is particularly prevalent in very preterm infants (VPI) due to the decreased surfactant synthesis by type 2 alveolar cells and secretion or its inactivation [73,74]. The identification of vitamin D receptors (VDR) in type II pneumocytes prompted researchers to investigate vitamin D’s role in pulmonary maturation and surfactant proteins and phospholipids production to determine if VDD is a risk factor for RDS and whether its effects can be reversed [65,75,76]. This highlights the vital role of the active form of vitamin D as a key influence on the lung, shaping its development during prenatal lung development. The research has demonstrated a correlation between maternal VDD and decreased lung volume and vital capacity, as well as increased airway muscle mass and reactivity in animal studies [77]. Abnormal lung histology, reduced angiogenesis, increased total respiratory system resistance, and elevated inflammatory factors have been linked to VDD in animal models of BPD [78,79]. Mandell et al. [80] noted persistent lung growth abnormalities in the offspring of a VDD animal model, including decreased alveolarization, increased vascular anomalies, and impaired lung function. Additionally, human studies have shown that vitamin D supplementation during lactation is associated with improved alveolarization and enhanced lung function [75].

A strong association between maternal vitamin D status and increased risk of RDS in preterm infants (*p* < 0.001) was recently noted in the meta-analysis performed by Boskabadi et al. [42]. Previous studies have found a link between lower levels of 25(OH)D in cord blood and an increased risk of developing RDS or acute respiratory complications [14,81,82]. Liu et al. [81] reported that a neonatal level of 25(OH)D is an independent risk factor for RDS development. Numerous previous studies identified VDD at birth, in the first 24 h of life, or at admission in the neonatal intensive care unit as a risk factor, or even as an independent risk factor, for RDS in preterm neonates [41,82,83,84,85,86,87,88,89]. Dogan et al. [87] found that higher neonatal VD status may play a protective role against RDS (risk ratio 0.089, *p* = 0.040). This suggests that maternal supplementation with vitamin D could be beneficial in reducing RDS incidence. However, the optimal timing and dosage for supplementation, as well as the ideal maternal vitamin D status, remain unknown.

Routinely monitoring serum levels of vitamin D in premature infants at birth and individualized vitamin D supplementation may become an important part of RDS management that could decrease the incidence and severity of this severe neonatal complication. Establishing a cut-off level for vitamin D at birth presents an opportunity for clinicians to optimize 25(OH)D levels in preterm infants. The research indicates that a 25(OH)D level of less than 50 nmol/L is a significant risk factor for RDS in this population, with an odds ratio (OR) of 0.195 and a *p*-value of 0.017 [41]. Additionally, Yi et al. [25] highlighted an increased incidence of RDS associated with severe VDD. Kim et al. [85] found that the OR for RDS escalates considerably as vitamin D levels decrease: OR = 3.478 (*p* < 0.001) for levels < 30 ng/mL, OR = 4.549 (*p* < 0.001) for levels < 20 ng/mL, and OR = 17.267 (*p* = 0.044) at levels < 15 ng/mL. Furthermore, lower serum 25(OH)D concentrations are significantly associated with an increased severity of RDS, including the need for surfactant administration, the duration of continuous positive airway pressure (CPAP) [86], mechanical ventilation duration [90,91], the duration of oxygen therapy, and the incidence of pneumothorax [91].

A recent meta-analysis and systematic review by Boskabadi et al. [42] comprising 1582 infants tried to evaluate the correlation between vitamin D status and the risk for developing RD, RDS, and transient tachypnea of the newborn (TTN). The research indicates that infants diagnosed with RD tend to have lower levels of 25(OH)D and the authors suggested that the serum 25(OH)D concentration may be considered a valuable respiratory biomarker. Additionally, a significant correlation has been identified between serum 25(OH)D levels and the risk of both RD and TTN. Maternal prophylactic vitamin D supplementation significantly reduced risk of RD, while vitamin D administration in preterm newborns notably lowered the incidence of respiratory complications [42]. The research has indicated important associations between lower 25(OH)D levels at birth and the incidence of respiratory complications in term neonates. Recent findings by Yangin Ergon et al. [92] established a connection between VDD and conditions such as TTN and RDS in infants born at term. Additionally, insufficient vitamin D status at birth was significantly linked to an increased risk of infectious pneumonia in term neonates (*p* < 0.05) [93], underscoring the critical role of vitamin D in neonatal lung health.

Providing adequate nutrition to preterm infants presents a challenge, particularly for those with BPD, as malnutrition can impair postnatal growth and reduce muscle strength [3]. The research examining the relationship between VDD and adverse respiratory outcomes, such as BPD, has produced varied findings, underscoring the complexity of this issue and the need for continued investigation [17,18,26,94]. A study by Cetinkaya et al. [40] demonstrated that the vitamin D status of mothers and preterm infants at birth plays a significant role in predicting the likelihood of BPD through univariable logistic regression. A meta-analysis performed by Park et al. [26] demonstrated that low levels of 25(OH)D at birth (*p* = 0.046) and VDD at birth (*p* = 0.007) are significantly linked to the development of BPD. Severe VDD, characterized by serum 25(OH)D level < 10 ng/mL, is linked to a higher incidence of BPD as compared to moderate VDD (serum levels between 10 and 20 ng/mL) (29.4% versus 8.7%) [38]. Other studies have confirmed a significant correlation between low vitamin D status at birth and an increased risk of BPD [45,89,95]. Additionally, the 25(OH)D level in the umbilical cord or at admission was identified as an independent risk factor for BPD [96]. Notably, in this study, each 1 ng/mL increase in vitamin D was associated with a 6% reduction in the risk of developing BPD. If the serum 25(OH)D concentration was <12.82 ng/mL, each 1 ng/mL increase in serum 25(OH)D resulted in an 87% reduction in the incidence of BPD, indicating a non-linear association between vitamin D status and BPD. Furthermore, a significant association between BPD and vitamin D was also found one month after birth by Yu et al. [97] in a retrospective study of 267 infants born before 32 weeks of gestation and with a BW of less than 1500 g. After demonstrating that infants with BPD had significantly lower mean 25(OH)D concentrations in their umbilical cords compared to those without BPD (11.6 versus 13.6 ng/mL; *p* = 0.016), follow-up results indicated that the incidence of BPD was significantly lower in preterm infants with serum 25(OH)D concentrations > 20 ng/mL (*p* = 0.024). Additionally, the authors found that vitamin D status is an independent risk factor for BPD (OR = 0.993), and identified a serum 25(OH)D level of 15.7 ng/mL as optimal for preventing BPD (area under the curve 0.585; sensitivity 75.4%; specificity 42.9%). These findings and the research conducted by Ge and colleagues in 2022 [98] strongly support the assertion made by Cui et al. [99] that vitamin D status in the umbilical cord should be recognized as a viable biomarker for BPD. However, multiple randomized controlled trials [6,51,98] failed to demonstrate that high doses of vitamin D supplementation (800–1000 IU) can prevent BPD in VLBW infants when compared to lower doses (200–400 IU).

In contrast, a meta-analysis showed no statistical association between 25(OH)D levels and BPD occurrence, irrespective of the dose of vitamin D used for supplementation (400 IU/day versus 800–1000 IU/day) [18]. Matejeck et al. [17] found no correlation between severe VDD and RDS or BPD, suggesting that GA was the only risk factor independently associated with RDS in their study. However, these results should be carefully interpreted as only appropriate-for-GA infants were included, the study size was small, a significant proportion of the included infants failed to complete the study, not all the maternal samples were available for analysis, and enteral supplementation with vitamin D3 began only after enteral feeding exceeded 150 mL/kg/day. The late initiation of enteral vitamin D supplementation could explain these conflicting results. In a more recent study by Tofe Valera et al. [19], no significant association was found between 25(OH)D levels < 20 ng/mL or >20 ng/mL and RDS and BPD in 50 preterm infants born at ≤32 weeks gestation and/or BW ≤ 1500 g monitored at 28 days and 4 months of life, despite the increased risk for RDS and BPD in preterm neonates with 25(OH)D levels < 20 ng/mL compared to those with vitamin D levels > 20 ng/mL. Notably, the mean 25(OH)D concentrations in the umbilical cord were higher than those reported in other studies (17.97 ± 9.35 ng/mL) and almost similar to those recorded at 28 days of life (17.82 ± 6.34 ng/mL) after administering vitamin D supplementation of 1000 IU/day via nasogastric tube starting at 48 h of life.

These findings suggest that the ideal regimen for vitamin D supplementation to support neonatal lung health may differ from that required for bone health. Therefore, further extensive studies with an adequate design are necessary to determine the optimal timing, dosage, and method of vitamin D administration, as well as to monitor and adjust vitamin D status in at-risk preterm infants.

## 4. Vitamin D and Associations with Infections

Sepsis, caused by a large variety of bacteria, viruses, and fungi, persists as a significant cause of morbidity and mortality during the neonatal period, with potential adverse effects on neurodevelopment. One of the most important risk factors for neonatal sepsis is prematurity [100,101].

The presence of vitamin D receptors in various immune cells—such as T and B cells, monocytes, macrophages, dendritic cells, and natural killer cells—has led to increased research into the clinical implications of vitamin D in inflammatory and autoimmune diseases, as well as infections. Studies have shown that these immune cells can express 1α hydroxylase, which allows them to convert 25-OH vitamin D into its active form, 1,25(OH)2 vitamin D (calcitriol) [102]. Mendelian randomized trials have demonstrated a correlation between certain autoimmune conditions and VDD [103]. Additionally, preclinical studies suggest that vitamin D can stimulate innate immune responses while inhibiting adaptive responses by regulating the transcription of several genes involved in immune function [104,105]. The influence of vitamin D on immune cell differentiation, maturation, and activation—specifically in T helper 1 and 2 cells [102]—is further evidence of its significant role in inflammation and infection. Vitamin D’s ability to induce antimicrobial peptides, such as cathelicidin and defensins [104,106,107], also supports this argument. Furthermore, vitamin D has been reported to obstruct viral replication, which contributes to viral clearance and reduces inflammatory responses associated with symptoms in viral infections. In a study examining the adult population admitted to an intensive care unit, vitamin D was found to be associated with an increased risk of severe infections, sepsis, and worse outcomes [108]. De Pascale et al. [109] and Cutuli et al. [49] observed that individuals with very low vitamin D levels experienced a longer duration of pneumonia and mechanical ventilation, as well as a greater need for vasopressors in cases of septic shock, when compared to those with vitamin D levels > 7 ng/mL. Zhou et al. [110] noted that adult patients with sepsis had lower serum 25(OH)D concentrations compared to those without sepsis. Furthermore, Li Y et al. [111] identified vitamin D as an independent risk factor for mortality in sepsis. Vitamin D deficiency was also linked to urinary tract infections during pregnancy [112].

A meta-analysis of 27 case–control and cohort studies involving infants and children with sepsis found that 25(OH)D levels were significantly lower in those with sepsis (*p* < 0.001). Additionally, the rate of sepsis was notably higher in patients with severe VDD (OR = 2.66, 95% CI 1.13–6.25, *p* < 0.001) [113]. In pediatric populations, there were several correlations between deficient or insufficient vitamin D status and respiratory tract infections, including COVID-19 [114,115]. A recent meta-analysis by Gan et al. [116] found that a 25(OH)D level < 20 ng/mL was significantly associated with urinary tract infections in children (*p* = 0.036). Marino et al. [117] connected a specific vitamin D polymorphism with increased severity of hepatitis B and higher viral load. Recently, Gao et al. [118] revealed that vitamin D3 has protective effects against respiratory syncytial virus infection, acting at the level of airway epithelial cells.

One of the first studies examining the implications of vitamin D during the neonatal period was conducted by Cetinkaya et al. in 2015 [119]. They compared 50 term neonates diagnosed with early-onset sepsis (EOS) to 50 neonates without EOS. The study found a higher incidence of severe VDD in infants with EOS, highlighting an association between low vitamin D status in both neonates and their mothers and the occurrence of EOS. These findings were confirmed by Kanth et al. [120], as serum 25(OH)D levels were significantly lower in term infants with EOS (*p* < 0.05); also, 35.9% of the infants diagnosed with EOS had a mean 25(OH)D level ≤ 12 ng/mL (*p* < 0.05). Vitamin D was evaluated as an adjuvant therapy alongside antibiotics in a study involving 30 term newborns with sepsis. These infants were compared to another group of 30 neonates who were treated with antibiotics alone. The researchers found that the infants with sepsis had significantly lower concentrations of 25(OH)D (*p* < 0.05), and VDD (defined as levels ≤ 20 ng/mL) was identified as a predictor of neonatal sepsis. Furthermore, vitamin D supplementation notably improved the sepsis scores and decreased high-sensitivity C-reactive protein (hs-CRP) levels at 3, 7, and 10 days of treatment (*p* < 0.05) [121]. A recent study involving both term and preterm infants found that both maternal and neonatal vitamin D levels were lower in those with sepsis than in the control group. Additionally, there was a significant correlation between maternal and neonatal vitamin D status and C-reactive protein, as well as all markers of sepsis in the blood count. The authors suggested that a neonatal 25(OH)D level < 20 nmol/L, alongside a maternal vitamin D level < 40 nmol/L, could serve as an early predictor of EOS [122]. Furthermore, Ergon et al. [92] recently confirmed a significant association between VDD and EOS in infants born at term.

A study conducted by Dhandai et al. [22] aimed to asses if VDD could be a risk factor for late-onset sepsis (LOS) in late preterm and term infants; the relationship between maternal and neonatal levels of vitamin D in a case–control study that included 120 infants divided into two groups, with or without sepsis, was investigated. The mean value of serum 25(OH)D in the sepsis group was significantly lower compared to controls (15.37 ng/mL versus 21.37, *p* = 0.001), and a significant risk for LOS was found in neonates with VDD (OR = 1.7 (95%CI 0.52–5.51); 25(OH)D concentrations were significantly lower in the mothers of septic infants compared to those of infants without sepsis (*p* = 0.004). Research has shown similar associations between lower 25(OH)D levels or VDD and EOS in preterm infants, as noted by Dogan et al. [87] and in VLBW infants, as noted by Czech-Kowalska et al. [123]. Baldan et al. [90] identified a 25(OH)D level ≤ 15 ng/mL as a predictive threshold for LOS in 112 preterm infants, of which 60% had serum 25(OH)D concentrations ≤ 15 ng/mL. Additionally, VDD was correlated with neonatal sepsis and was associated with mortality, need for ventilator support, longer hospital days, and positive blood cultures in both term and preterm infants [124]. In a study of 50 preterm infants with GA ≤ 32 weeks and/or a BW ≤ 1500 g, Tofe Valera et al. [19] found no significant differences in comorbidities, including LOS, between preterm infants with 25(OH)D levels < 20 ng/mL and those with levels > 20 ng/mL. Notably, while these infants received daily supplementation of 1000 IU of vitamin D, the rate of VDD was not reduced until 4 months after birth, and 90% of the infants with LOS had vitamin D levels < 20 ng/mL. Furthermore, 25(OH)D concentration at 28 days of life was identified as an independent risk factor for LOS. In term infants, insufficient vitamin D status was linked with an increased risk for infectious pneumonia, sepsis, and cytomegalovirus infection (*p* < 0.05) compared to those with sufficient 25(OH)D levels.

In a group of 619 newborns, including 292 preterm infants born at less than 27 weeks gestation, vitamin D status was evaluated after 2–4 weeks of vitamin D supplementation and no difference in the incidence of sepsis was observed among infants with deficient, insufficient, and sufficient vitamin D levels [25]. Abdel-Hady et al. [125] reported that interleukin 6 and tumor necrosis factor α concentration declined at 1 week of age and at the time of discharge following vitamin D supplementation with 400 versus 800 IU per day; however, no significant difference was found between the two dosage groups. Moreover, no significant difference was found for the duration of antibiotic therapy.

Further research, focusing specifically on preterm infants, is needed to determine the vitamin D threshold associated with an increased risk for neonatal sepsis (both EOS and LOS) and to identify the appropriate vitamin D supplementation regimen (timing, dose, monitoring, and dose adjustments). More studies are also required to confirm the relationship between maternal vitamin D status, as well as to investigate whether implementing a vitamin D screening and supplementation protocol during pregnancy could help prevent neonatal EOS.

## 5. Vitamin D and Retinopathy of Prematurity

Retinopathy of prematurity (ROP), a vasoproliferative disease with a multifactorial etiology that affects most of the VPI, remains the leading cause of neonatal visual loss impairment [126,127]. Numerous studies have investigated ROP development’s mechanisms and risk factors [126,127,128]. Researchers have explored the potential link between VDD and the severity of ROP, given vitamin D’s anti-inflammatory, immunomodulatory, antioxidant, and anti-angiogenic properties [129].

Since vitamin D receptors (VDR) and 1α hydroxylase were identified in the retina, the possible effects of 25(OH)D on the retina and its angiogenesis have been investigated. Jamali et al. [130] demonstrated that the inhibitory effect of vitamin D on retinal neovascularization depends on VRD expression and hypothesized that vitamin D plays an important role in retinal development and maturation through VDR. The same group showed that vitamin D and its receptors modulate retinal angiogenesis via the vascular endothelial growth factor (VEGF), particularly under hypoxic and hyperoxic conditions [131]. Vitamin D may exert anti-inflammatory effects, thus limiting the retinal and optic nerve damage; additionally, the antioxidant properties of vitamin D could reduce the retinal oxidative stress. The antiangiogenic, anti-inflammatory, and antioxidant effects are the proposed mechanism for the protective role of vitamin D for the developing eye and visual system [127,132]. Another group of researchers studied the relationship between vitamin D (25(OH)D2 and 25(OH)D3) and VEGF in tears collected from preterm infants with ROP of various severity. The authors found that vitamin D regulates VEGF in an oxygen-dependent manner as they demonstrated a positive correlation between vitamin D and VEGF with ROP stage 1 (an early stage when hypoxia dominates) and a negative correlation with ROP stage 3 (severe ROP in a period characterized by hyperoxia) [133].

In a prospective study from Turkey including 97 preterm infants, 73.2% developed ROP and had significantly lower serum 25(OH)D concentrations (*p* < 0.001) than those without ROP. However, the study has a few limitations: the maternal level of 25(OH)D was not assessed, nor was the vitamin D status of the preterm infants after supplementation was initiated, and the cut-off values used for defining deficiency or sufficiency of vitamin D were slightly different than the most frequently used, as levels ≥ 16 ng/mL were considered normal [128]. A retrospective study by Kim et al. [89] reported VDD at birth (<20 ng/mL) as independent risk factor for ROP (OR = 5.49 (95% CI 1.16–26.00), *p* = 0.003). A significant difference was found in ROP incidence among infants based on their 25(OH)D levels at birth: 32.5% of infants developed ROP with levels < 10 ng/mL compared to 16.4% at levels between 10 and 20 ng/mL and 5.3% with > 20 ng/mL (*p* = 0.001). Notably, the authors reported very high rates of severe VDD (<10 ng/mL; 44.1%) and overall VDD (79.8%). Vitamin D supplementation was individualized based on their vitamin D status at birth: 400 IU per day in preterm infants with sufficient vitamin D, 1200 IU per day for those with severe VDD, and 400 to 800 IU for the others. A case–control study focusing on preterm infants born before 34 weeks of gestation and weighing < 2000 g, supplemented with 100 IU vitamin D per day from the second week of life, reported GA, BW, and vitamin D status as significantly associated with ROP; also, VDD persisted at 4 weeks of life in preterm infants that developed ROP [134].

Boskabadi et al. [135] found significant differences in both maternal (*p* = 0.015) and neonatal (*p* < 0.001) levels of 25(OH)D between the premature infants who developed ROP and those who did not. They also reported a negative association between VDD and the severity of ROP [135]. In a recent study by Yin et al. [127] including 217 preterm infants (<32 weeks of gestation), ROP incidence was correlated with lower BW and GA, sepsis, long-term need for non-invasive and invasive respiratory support and oxygen therapy, and 25(OH)D levels. In multivariate regression, GA, BW, mechanical ventilation duration, vitamin D status, and sepsis were identified as independent risk factors for ROP. Furthermore, serum 25(OH)D concentrations at one month of life were significantly lower in the ROP group compared to the non-ROP group (*p* < 0.001) despite infants receiving vitamin D supplementation of 400 IU per day.

Our understanding of the relationship between vitamin D status at birth and postnatal supplementation is improving as more studies emerge on this topic. Both experimental and clinical research are needed to clarify how we can effectively use this inexpensive treatment to prevent ROP, a serious and debilitating condition associated with extreme and very preterm birth.

## 6. Vitamin D and Necrotizing Enterocolitis

Despite extensive research, NEC continues to be one of the most severe and life-threatening complications associated with prematurity [49]. Several studies indicated that VDD is linked with impaired intestinal barrier function, suggesting that adequate vitamin D status could offer protection against injury to the intestinal epithelium, potentially reducing the incidence of NEC. Vitamin D reduces intestinal permeability and tumor necrosis factor α expression, thereby contributing to tight junction restoration and attenuation of the intestinal inflammation [18,136,137]. The regulation of intestinal stem cell activity has also been proposed as a protective mechanism by which vitamin D may alleviate gut lesions [136]. The activation of toll-like receptor 4 (TLR4) plays a key role in NEC development through reduced proliferation and increased apoptosis. Vitamin D exerts one of its immunomodulatory effects by regulating TLR4 [138]. Barut et al. [139] explored the genetic role of VDR gene polymorphism in NEC and demonstrated that Fok 1 C > T polymorphism increases NEC risk by 2.697 times, while TT polymorphism is associated with a 4.5-fold higher risk of NEC (*p* = 0.033).

Studies in adults demonstrated an association between insufficient vitamin D and chronic inflammatory intestinal disease through its potential to regulate the innate and adaptative immune system, protect and maintain the intestinal barrier, and influence the intestinal microbioma [140]. In a study group of 82 children aged 1–3 years with invasive enteritis, Yang et al. [141] found significantly lower levels of 25(OH)D compared to 90 healthy children of the same age (*p* < 0.01). Interestingly, exclusive breastfeeding, an increased duration of outdoor time, and regular vitamin D supplementation were associated with a lower risk of invasive enteritis.

The study by Cetinkaya et al. [142] was the first that investigated the potential connection between maternal and neonatal vitamin D status and the risk of NEC in preterm infants. Apart from reporting that the preterm infants with NEC had significantly lower GA and BW, higher rates of persistent ductus arteriosus, intraventricular hemorrhage (IVH), ROP, BPD, and an increased need for respiratory support, the authors noted that both the infants diagnosed with NEC and their mothers demonstrated significantly lower levels of 25(OH)D compared to the control group (*p* = 0.001, 0.004, respectively). Additionally, severe VDD was found in 80.8% of the preterm infants with NEC compared to 53% of those without NEC. Notably, none of the preterm infants with NEC had sufficient levels of serum 25(OH)D (>32 ng/mL). The risk of NEC was reduced by 0.86 times for each 1 ng/mL increase in the mother’s 25(OH)D level (OR = 0.858, 95% CI 0.765–0.962, *p* < 0.009). Additionally, a sufficient neonatal 25(OH)D level was reported to reduce the NEC incidence by 0.9 times (OR = 0.891, 95% CI 0.821–0.966; *p* < 0.005) in the univariate analysis; however, this effect was not confirmed in the multivariate analysis [142].

In a mixed study involving both preterm infants and a rat model of induced NEC, Shi et al. [143] reported that preterm infants that developed NEC had significantly lower 25(OH)D levels (33.5 ± 3.58 versus 52.7 ± 5.99 nmol/L) compared both to healthy preterm infants and term neonates. The authors found elevated expression of TLR4 and suppressed VDR expression in intestinal epithelium in the NEC experimental model. Moreover, vitamin D treatment of the animal model attenuated intestinal apoptosis and inflammation, protecting the gut barrier and function, and partially suppressed VDR inhibition. However, the study sample size enrolled in this study was small. The comparison between preterm infants with GA < 36 weeks with and without NEC revealed that both maternal and neonatal serum 25(OH)D were lower in the NEC group as compared to the non-NEC one (*p* < 0.001). Logistic regression showed that GA, BW, and maternal and neonatal 25(OH)D were associated with NEC (*p* < 0.05) in a study by Yang LR et al. [144].

In contrast, Tofe Valera et al. [19] found no difference in the NEC rates between preterm infants with 25(OH)D < 20 ng/mL and those with 25(OH)D levels > 20 ng/mL (8% versus 8%; RR = 0.92), evaluated at 28 days of life. Similarly, another study found no significant difference in NEC prevalence between preterm infants with severe VDD, VDD, insufficient, and sufficient vitamin D status, evaluated at 2 and 4 weeks of life [25]. Consequently, more comprehensive studies are essential to determine whether sufficient levels of 25(OH)D can lower the incidence of NEC, a significant comorbidity in preterm infants, ultimately enhancing their survival rates. The effectiveness of maternal vitamin D status screening and supplementation in preventing NEC is another potential field that needs evaluation.

## 7. Vitamin D and Neurodevelopment

By regulating neural cell proliferation, differentiation, and survival in a developing brain, vitamin D, a neurosecosteroid hormone, exerts important effects on brain health and functions starting in fetal life [145,146]. Most evidence regarding the relationship between vitamin D and children’s brain health originates from studies performed during pregnancy. In a study comprising 1650 mother–child pairs, Morales et al. [147] demonstrated that, after adjusting for cofounders, for each 10 ng/mL increase in the maternal 25(OH)D level there is an 11% reduction in the total number of attention deficit-hyperactivity disorder (ADHD) symptoms in the offspring. Moreover, higher maternal 25(OH)D concentrations were associated with a decreased risk of ADHD. These findings were confirmed by Sucksdorff et al. [148] in a nationwide populational case–control study; lower maternal vitamin D status was, again, associated with ADHD in the offspring, even after adjusting for socio-economic status and maternal age. A meta-analysis comprising 25 trials also evaluated the effects of maternal vitamin D at birth on the child’s neurodevelopmental outcome. The lowest maternal 25(OH)D concentrations during pregnancy were significantly associated with a summed risk of ADHD (RR = 0.72, *p* = 0.002) and autistic traits (RR = 0.42, *p* = 0.001). In contrast, a slight protective effect of higher levels of maternal vitamin D was found for language development and behavioral disturbances; the authors concluded that an increase in prenatal exposure of the fetus to vitamin D might improve cognitive abilities and reduce the risk of autism traits and ADHD [149]. Lee et al. [150] noted an association between both maternal and neonatal low vitamin D status and autism; the risk for autism was 1.75 times higher at vitamin D levels under the median compared to levels above the median (95% CI 1.08–2.86), even after adjusting for cofounders. A meta-analysis by Tous et al. [34] reported that maternal 25(OH)D levels < 50 nmol/L during pregnancy were associated with lower mental and language scores at follow-up (nonsignificant). These results differ from the secondary analysis of a randomized controlled study by Sass et al. [151], in which no effects were found after vitamin D supplementation during pregnancy with 2800 IU versus 400 IU on motor, cognitive, global neurodevelopment, emotional, and behavioral problems in 551 children evaluated at 6 years of age.

Two interesting studies from Denmark demonstrated an association between low neonatal vitamin D status at birth—evaluated on dried blood spots—with diminished cognitive abilities in adolescence using a nested birth cohort [152] and between increased vitamin D concentrations at birth and an increased risk for epilepsy diagnosed at 1 to 4 years of age in a case–cohort study [153]. Interestingly, in a review on the effects of vitamin D, Grant et al. [145] also mentioned a U-type curve relationship between vitamin D status—between too low and too increased—and lower cognitive scores.

Recently, Sammallahti et al. [154] reported a link between low maternal vitamin D status during pregnancy and increased negative affectivity during infancy. In 546 infants randomized to receive 1200 IU versus 400 IU vitamin D from 2 weeks of age up to 2 years, Sandboge et al. [155] reported a decrease in the frequency of internalizing problems. In contrast, in another Finish randomized trial, no association was found between vitamin D levels and the primary neurodevelopmental outcomes evaluated using Ages and Stages Questionnaire and Infant–Toddler Social and Emotional Assessment at 2 years of age after THE randomization of term newborns to 1200 IU or 400 IU vitamin D starting at 2 weeks of age [156]. However, 92% of the study group had sufficient vitamin D at birth.

In preterm infants, Fort et al. [51] assessed neurological problems during hospitalization and the neurodevelopmental outcome in extreme premature infants (23–27 weeks of gestation) randomized to receive no vitamin D supplementation or 200 IU or 800 IU vitamin D. No difference was observed between groups in terms of IVH (each grade and all grades), seizures, and neurodevelopment at 2 years. Similarly, Tofe-Valera et al. [19] observed no difference in the IVH and periventricular leukomalacia (PVL) rates between preterm infants with 25(OH)D levels < 20 ng/mL compared to those with levels > 20 ng/mL. Neither IVH nor PVL were associated with vitamin D status at birth or at 28 days of life. No significant difference between the morbidities and complications associated with prematurity, including IVH, was observed in preterm infants at ≤32 weeks of gestation, with 25(OH)D levels < 15 ng/mL compared to >15 ng/mL at birth (*p* = 0.07) according to Baldan et al. [90].

In 70 of 80 preterm infants born at 23–27 weeks of gestation, followed up in a study by Salas et al. [43] at 2 years, no significant differences were found between the cognitive scores of the infants with no supplementation and those supplemented with 200 IU or 800 IU vitamin D (*p* = 0.15). However, there was a tendency toward increased cognitive scores and language development and reduced neurodevelopmental impairment in former preterm infants supplemented with higher vitamin D doses. Vitamin D deficiency and insufficiency were associated with significantly lower scores in five areas of the Gessel scale at 10–18 corrected age (CA) (*p* < 0.05) in 161 late-preterm infants. Additionally, the language scores were lower at 10 months CA and the motor scores were lower at 18 months in vitamin D-deficient infants (*p* < 0.05). Furthermore, individualized supplementation with 800 IU vitamin D led to increased adaptative ability at 10 and 18 months CA [157]. Recently, Guo et al. [158] demonstrated a positive correlation between 25(OH)D levels with maternal supplementation with vitamin D during pregnancy and neonatal postnatal supplementation (*p* < 0.05). Moreover, the vitamin D status of the infants at 42 days of life and at 3 months were correlated with the developmental quotient (DQ) at 6, 9, 12, and 18 months (*p* < 0.05). Notably, the DQ was significantly lower in former preterm neonates with VDD at 42 days and persistent VDD at 3 months (*p* < 0.05).

Interestingly, both maternal prenatal vitamin D status and neonatal vitamin D status do not appear to affect short-term neurological outcomes, such as IVH, PVL, or seizures. However, there is a growing body of evidence linking both low and high 25(OH)D levels to medium- and long-term neurodevelopmental outcomes, including cognitive, motor, behavioral, and language development. Further research is needed to define the optimal supplementation dose of vitamin D, particularly in extremely preterm infants, the population at greatest risk for neurodevelopmental impairments. Exploring optimal maternal vitamin D supplementation offers a valuable opportunity to enhance neurodevelopment in both preterm and full-term newborns.

## 8. Other Potential Implications of Vitamin D in Neonatal Period

### 8.1. Vitamin D and the Kidney

The active form of vitamin D, 1,25(OH)_2_D, is produced from 25(OH)D in the kidneys through hydroxilation. Renal failure has been linked to decreased levels of 25(OH)D [23]. Studies in both adults and children have reported various associations between VDD and acute kidney injury (AKI) [159,160], chronic kidney injury [160], renal scarring related to recurrent urinary tract infections [161], and Fanconi syndrome [162]. In animal studies, pretreatment with pericalcitol, a synthetic analog of calcitriol, reduced the levels and expression of matrix metalloproteinases, thereby protecting the renal tubules from ischemic reperfusion injury resembling AKI in a rat model [163].

Conflicting results regarding the prenatal effect of VDD on the number and maturation of nephrons were reported in experimental models [164]. One study examined the impact of nutritional intake during pregnancy on fetal development and found that maternal low levels of 25(OH)D at mid-pregnancy were associated with the calculated creatinine clearance but not with the calculated cystatin C clearance in offspring at school age (*p* < 0.05). Furthermore, maternal 25(OH)D status was also associated with blood levels of creatinine in children but not with the cystatin C blood levels in offspring (*p* < 0.05). The authors interpreted these findings as evidence that maternal vitamin D status during pregnancy may influence kidney structure and function [165]. To our knowledge, no studies in preterm or term infants evaluated the effects of VDD on kidney function or renal outcomes in the short or long term.

Further research is needed to enhance our understanding of how inadequate vitamin D intake during pregnancy may affect kidney development, maturation, and function.

### 8.2. Vitamin D and Jaundice

Hyperbilirubinemia is a common condition during the neonatal period, and is usually benign if there are no factors that could lead to a dangerous increase in bilirubin levels. Severe hyperbilirubinemia can result in serious complications, such as bilirubin encephalopathy and kernicterus, with dramatic consequences for the infant’s neurodevelopment and increased risk of death. Vitamin D metabolism involves the hepatic conversion of 7 dehydrocholesterol (provitamin D) into the 25(OH)D. Biswas et al. [166] recently reported significantly lower mean 25(OH)D levels (*p* = 0.001) and a high prevalence of VDD in 60 infants with cholestatic jaundice, with 60% of these infants diagnosed with biliary atresia.

Asemi et al. [167] investigated the effect of maternal supplementation with vitamin D during pregnancy; their analysis showed that the infants born to mothers who received vitamin D supplements experienced jaundice less frequently compared to those born to mothers in the placebo group (no vitamin D supplements) (27.3% versus 60.9%, *p* = 0.02). In a cohort study on 304 pregnant women enrolled before 20 weeks of pregnancy, a higher maternal vitamin D status and vitamin D binding protein levels were significantly associated with lower bilirubin levels. However, the association became insignificant after adjustment for the cofounders [168].

Recently, several studies reported on a possible association between VDD and neonatal hyperbilirubinemia [169,170,171,172,173]. All studies were small and involved term infants, except the case–control trial by Al Banna et al. [169], which included a few late preterm infants, and all, except the study by Mehrpisheh et al. [173], reported lower or significantly lower vitamin D levels in jaundiced neonates compared to controls. The recent meta-analysis by Huang et al. [174], which included six case–control studies and 409 cases, demonstrated that the mean 25(OH)D level was 7.1 ng/mL lower in infants with hyperbilirubinemia compared to the control group (*p* < 0.05). Moreover, if only infants with serum bilirubin values between 15 and 20 mg/dL were considered, the mean 25(OH)D value was 9.52 ng/mL lower than in controls (*p* < 0.05). Polymorphisms of the vitamin D metabolic pathway genes could be incriminated, at least in some cases, for the association between VDD and hyperbilirubinemia, according to Zhou et al. [175]. The authors identified the genotype rs12785878 in cases associated with both severe VDD (<15 ng/mL) and severe hyperbilirubinemia.

Other authors reported on the effect of phototherapy with or without vitamin D supplementation (400–800 IU/day) in term infants with neonatal bilirubinemia. Some studies reported a beneficial effect of vitamin D supplementation (reduced bilirubin levels and/or duration of phototherapy, reduced hospitalization days) [169,171,176]. However, other studies found no significant effects [177].

Hyperbilirubinemia can have severe consequences for preterm infants as prematurity is associated with various factors that may compromise the blood–brain barrier. If a connection exists between VDD and an increased risk for hyperbilirubinemia, it is a question that warrants investigation, as well as the potential of vitamin D supplementation to reduce bilirubin concentrations in blood.

### 8.3. Vitamin D and Mortality

In the study by Mori et al. [178] including 63 preterm infants with GA < 34 weeks and BW < 1500 g monitored up to the age of 1, no significant differences were found between infants with and without significant morbidities regarding the 25(OH)D level in the umbilical cord. Similarly, Matejek et al. [19] showed that 25(OH)D concentration < 25 nmol/L was not correlated with the clinical outcomes of prematurity.

There are currently no studies specifically focused on the relationship between VDD and mortality in preterm or term neonates, yet some authors reported observations on this possible association. A study on 40 preterm infants found no correlation between vitamin D status and mortality [179]. Abdel Hady et al. [125] showed no significant difference in mortality between two groups of preterm infants with GA ≤ 28 weeks supplemented with daily 400 or 800 IU of vitamin D. Additionally, the research conducted by Xi et al. [25] included 619 preterm infants, 292 of them born < 27 weeks gestational age, and reported no difference in mortality based on 25(OH)D levels, categorized as severe deficiency, deficiency, insufficiency, or sufficiency. The only study that reported an association between VDD and mortality involved a group of 41 infants, both term and preterm, who were diagnosed with sepsis [111].

## 9. Vitamin D: Recommendations for Supplementation

The traditional concepts and current guidelines for assessing vitamin D status and optimal supplementation warrant careful reconsideration. Recent research has enhanced our understanding of vitamin D’s protective role and effects. Additionally, some researchers argue that the normal vitamin D values traditionally applied to children and adults may not adequately account for the needs of premature infants [36,39,51,106,107].

Adnan et al. [138] reported a positive correlation between 25(OH)D levels and BW (r = 0.23, *p* = 0.03) and GA (r = 0.447, *p* < 0.001); 18% infants, 40% of them born before ≤28 weeks, had serum 25(OH)D levels at birth consistent with VDD and 35% had insufficient levels of vitamin D. Stronger correlations were observed by Budhwar et al. [24]: 25(OH)D levels were correlated with GA with r = 0.9368, *p* < 0.0001 and with BW with r = 0.9559 and *p* < 0.001 in the comparison between preterm and term infants. Moreover, a significant correlation between 25(OH)D and GA and BW was also noted by Ardastani et al. [86], while Choudhury et al. [180] identified an association between VDD and lower GA and length. However, other studies found insufficient evidence to support a relationship between 25(OH)D levels and GA or BW [21,179,181,182]. Park et al. [183] showed that 91.7% of preterm newborns had VDD at birth, while 51.1% had severe VDD at birth. No correlation between severe VDD and early/moderate or late prematurity was identified. Also, 25(OH)D serum concentrations were significantly increased during autumn compared to other seasons (*p* = 0.013).

Despite the significant prevalence of VDD in preterm infants, there is still a lack of consensus on the optimal levels and daily intake requirements for supplementation. This uncertainty presents challenges in ensuring that the specific needs of this high-risk population are adequately met [18,39]. Previously, different strategies to prevent and treat VDD were proposed based on various literature sources and guidelines worldwide. However, the lack of standardization regarding the timing, dose, formulation, and method of administration of vitamin D supplementation in preterm neonates is challenging and hinders clear conclusions from being drawn from the available studies, as various protocols are used by researchers in clinical trials (Table 2).

For preterm infants, the current recommendations for vitamin D supplementation dosage vary worldwide, ranging from 400 to 1000 IU per day [4,6,61,214] (Table 3). Most of these recommendations primarily focus on bone density and mineralization, aiming to prevent metabolic bone disease or rickets.

The effectiveness of different dosing regimens for vitamin D supplementation in premature infants was evaluated in several studies to identify the optimal dose (Table 2). These studies also examined the best timing for initiating enteral supplementation [8,9,18,51,198]. A recent randomized controlled trial (RCT) investigated the safety and benefits of different regimens (200 IU in the parenteral nutrition/day versus 400 IU/day versus 1000 IU/day) used for enteral vitamin D supplementation in extremely premature infants. The study found important and relevant significant differences in 25(OH)D levels measured at 14 days of life (*p* < 0.0001) and at 28 days of life (*p* < 0.001) among the three dosing groups, with the highest dose being the most effective. The results suggest that the current AAP recommended dose of 400 IU/day for vitamin D supplementation is inadequate for premature infants. A higher dose was safe and may help improve VDD by 2 weeks of life, even before achieving full enteral feeding [8].

Preterm infants require higher doses than term infants, as the evidence indicates that higher doses (800–1000 IU/day) are associated with better immune function, improved postnatal growth, and a faster improvement in VDD by the 14th day of life. Also, no toxicity reports were mentioned that were related to the usage of higher doses of vitamin D [8,9,18,51].

Vitamin D can be provided through either parenteral nutrition or oral administration. In the Adnan et al. [138] study, parenteral nutrition was initiated from the 1st day of life and provided at 120 IU/day for preterm infants with a BW < 1000 g and 260 IU/day for those weighing 1000–3000 g. However, the parenteral administration of vitamin D is not available worldwide. Giustina et al. [39] recommended against parenteral administration as it is less efficient compared to enteral administration. Moreover, daily versus periodic administration of vitamin D supplements is recommended, in an individualized manner based on the specific needs of different population groups, body weight, ethnicity, cutaneous pigmentation, and latitude, which are all factors affecting 25(OH)D levels.

The effectiveness of oral daily intake versus the intramuscular administration of vitamin D was also evaluated [216]. Numerous studies have demonstrated that both oral vitamin D supplementation and intramuscular injections can increase 25(OH)D levels. However, oral vitamin D supplementation results in a faster rise in these levels. Kashaki et al. [10] showed that 25(OH)D levels were significantly increased in the group receiving oral drops of vitamin D, 1000 IU/day compared to those who received a single intramuscular dose of 15,000 IU (*p* = 0.006). Notably, although all preterm infants received an intramuscular dose of 300,000 IU within the first few days of life, vitamin D supplementation was started only from the 16th day of life.

At present, there is no consensus regarding the optimal timing for the initiation of the enteral supplementation of vitamin D in preterm infants, usually provided as cholecalciferol (Vitamin D3) [8,138]. The timing of vitamin D supplementation varies considerably across different studies (Tabel 2). To our knowledge, none of the studies initiated enteral supplementation with vitamin D from birth.

In a study conducted by Matejek et al. [17], enteral supplementation with vitamin D3 began only after enteral feeding exceeded 150 mL/kg/day, while, in other studies, enteral supplementation was started within the first week of birth using multivitamin drops via orogastric tube [8,51]. Other studies report the initiation of oral vitamin D supplementation with a multivitamin drop providing 400 IU vitamin D3 after the 14th day of life or when parenteral nutrition ceased [138] (Table 2).

The prolonged use of parenteral nutrition and insufficient intake of breast milk significantly increased VDD risk in VPI. Cho et al. demonstrated that a low intake of breast milk and long-term parenteral nutrition are significant risk factors for VDD [46]. In a study conducted by Munshi et al. [198], it was found that more than 80% of preterm infants, despite receiving 400 IU/day of oral vitamin D, still exhibited deficient or insufficient levels of 25(OH)D after 4 weeks of supplementation. Enteral supplementation was initiated when feeding volumes reached 140 mL/kg/day. Since parenteral nutrition, fortified mothers’ own milk, and enriched special formula cannot provide adequate 25(OH)D levels, initiating oral supplementation soon after birth is essential to correct VDD in preterm infants [198], as this approach may improve clinical outcome associated with VDD.

Most studies reported initiating oral vitamin D therapy based on the feeding volume (Table 2), frequently when total enteral feeds reached ≥120 mL/kg/day [9]. An analysis of the literature suggests that early intervention for the optimal enteral supplementation of vitamin D is not only successful in achieving higher 25(OH)D status at one month but is also linked with improved outcomes [9,47]. However, it remains unclear when or how early the vitamin D supplementation should begin, or whether pure vitamin D drops or multivitamin drops should be used.

The enteral supplementation of vitamin D was administered in various modes: drops in the mouth, using a small syringe, on nasal or oral gastric tubes, before feeding or mixed with milk, formula, or fortifier (Table 2). The infusion systems used for feeding are associated with a significant loss of lipids. Long-term debates on lipids and lipid-soluble vitamins waste on the walls of the plastic tubes or syringes used for preterm feeding have come to an end, as effective strategies to reduce these losses were identified: the use of syringes with eccentric adaptor tip, peristaltic pumps, frequent milk homogenization, and the use of silicon tubes and syringes. Moreover, the infusion rate does not impact the transfer of lipids [217,218]. Mixing vitamin D formulations with human milk, formula, or fortifiers raises concerns related to the adverse effects of the increased osmolality (such as necrotizing enterocolitis), but there is no evidence of this, as none of the studies using these approach for vitamin D supplementation reported adverse effects.

There is a pressing need for consensus guidelines that specify the cut-off values for determining sufficient, deficient, or insufficient vitamin D levels in preterm infants. Additionally, guidelines should define the optimal dose for each high-risk category of premature infants, the timing of the initiation of enteral vitamin D supplementation, and the optimal product.

Experts are concerned about the unsatisfactory results of the randomized controlled studies on vitamin D supplementation. The number of participants, their various levels of exposure to vitamin D, the measurements of vitamin D status at birth in direct correlation with maternal vitamin D status and/or supplementation with vitamin D, the postnatal protocol for vitamin D supplementation in terms of timing, dose, duration and monitoring, and biases in conduct and/or analysis may lead to inconclusive or conflicting results. These experts argue that studies on vitamin D’s associations with disease should be focused on its potential extraskeletal benefits, not only on bone health, in all groups of population. In this respect, observational studies seem more appropriate as these can treat vitamin D as a nutrient, with cut-off levels for adverse effects, and not as a drug [36,106,107,219]. Furthermore, Gospodarska et al. [220] suggest that future studies should segregate patients based on their genetic responsivity to vitamin D supplementation (high, medium, and low responsivity) to conduct a genomic evaluation of individuals with low response to high doses of vitamin D. This approach may also fit the population of preterm neonates, an inhomogeneous, vulnerable group of infants.

## 10. Conclusions

For decades, vitamin D has played a crucial role in the prevention of bone demineralization and metabolic bone disease management. Its significance in maintaining skeletal health is well-established and it continues to be a vital component of international nutritional guidelines. Recently, research has revealed additional effects of VDD in preterm infants, linking it to multiple health conditions. Despite the growing interest in vitamin D research, there is still uncertainty regarding clear recommendations for preterm infants concerning the optimal dosage, type of product, and timing for initiating vitamin D supplementation to prevent VDD, as well as in screening for VDD, monitoring protocol, and the continuous adjustment of dosage to maximize benefits.

Preterm infants are particularly susceptible to VDD. It is well established that neonatal vitamin D levels are influenced by the vitamin D status of their mothers; inadequate maternal levels can negatively affect infant health. Therefore, it is crucial to implement strategies to prevent VDD in pregnant women by developing clear recommendations for screening and supplementing vitamin D in pregnant and lactating mothers.

Further exploration is essential to unlock the potential benefits of assessing maternal vitamin D status during pregnancy and/or at birth. Additionally, whether screening for VDD could serve as a low-cost intervention that impacts preterm delivery rates or severe complications associated with prematurity, such as RDS, BPD, sepsis, NEC, ROP, neurodevelopmental impairment, or impaired postnatal growth, should be explored. Larger studies, with an appropriate design and standardized definitions, are required to develop effective strategies for screening and supplementing vitamin D in preterm infants. These studies should thoroughly investigate whether specific GA or BW cut-off values for defining VDD can be identified, and determine whether the 25(OH)D level should be monitored even after one month postnatal age until a normal level is reached.

## Figures and Tables

**Table 1:** Classification of vitamin D status by serum 25-OH vitamin D cut-off values as recommended by various expert panels [4,5,6,7].

Vitamin D Status	Institute of Medicine (IOM)		Pediatric Endocrine Society (PES)		Endocrine Society		American Academy of Pediatrics (AAP)	
	ng/mL	nmol/L	ng/mL	nmol/L	ng/mL	nmol/L	ng/mL	nmol/L
Deficiency	<12	<30	<15	<37.5	<20	<50	<15	-
Insufficiency	12–20	30–50	15–20	37.5–50	20–29	50–72.5	15–20	40–50
Sufficiency	>20	>50	>20	>50	≥30	≥75	>20	>50
Excess	>100	>250	>100	>250	-	-	-	-

**Table 2:** Enteral vitamin D supplementation in preterm infants: recent trials.

Initiation of enteral vitamin D supplementation	On the 2nd day of life [21].
	At 48 h of life [98], if feasible [19].
	On the 7th day of life [184].
	On the 7th day of life or when full enteral feeding is achieved [185,186,187].
	At approximately 1 week of life, after achieving the target Ca/P supplementation in PN and stabilization of oral feeding post the early trophic feeding period [188].
	On the 8th day of life [41].
	On the 14th day of life if enteral feeding is tolerated [189].
	From the 15th day of life [97].
	After the 14th day of life if parenteral nutrition is stopped (parenteral nutrition is interrupted at 120 mL/kg/day) [138].
	At 30 mL/kg/day of enteral feeding [190].
	With the initiation of enteral feeding, in addition to parenteral supplementation [191].
	Starting at a minimum of 75% of the full enteral feeding in the first 2 weeks of life (median 9 days of life) [192].
	On the first day when full enteral feeding is achieved [193,194].
	At 100 mL/kg/day enteral nutrition [195,196].
	At 100 mL/kg/day enteral feeding if no evidence of sepsis exists [197].
	After full enteral feeding is achieved; cessation of the parenteral feeding at 100 mL/kg/day [71].
	When at least 100 mL/kg/day enteral feeding is reached (7 +/−3 days of life) [180,181].
	At 140 mL/kg/day enteral feedings [198].
	After reaching >150 mL/kg/day enteral feeding [20].
	At >120–150 mL/kg/day enteral feeding (4/35 respondents to the survey) [199].
	At 160 mL/kg/day 20.1 ± 15.5 days of life [200].
	Based on feeding volume—56% of the respondents; when a minimum of 120 mL/kg/day of minimal enteral feeding is reached—71% of the respondents; first week of life—34.8% of the respondents to the survey; in the second week of life—19% [9].
Vitamin D dose at the initiation of enteral supplementation	100 IU/kg/day [89].
	200 IU/day [155].
	200 vs. 800 IU/day [8,123].
	400 IU/day [71,138,185,186,188,194,195,196,198,201,202,203,204,205,206,207].
	400 IU/day—77% of the respondents to the survey [9].
	400–500 IU/day—most of the respondents to the survey, 900–1000 IU/day—2/35 respondent units [199].
	400 versus 800 IU/day [191,208].
	400 versus 800 versus 1000 IU/day [192].
	500 IU/day [20].
	600 IU/day [21].
	At a median of 609 IU/day (519–678) [209].
	700 IU/day [41].
	800 IU/day [97,98,180].
	800–900 IU/day [189]
	1000 IU/day [19,210].
Vitamin D formulation	Vitamin D solution [21,51,204].
	Syrup with vitamin D, calcium, phosphorus, and syrup containing vitamin D or drops of vitamin D solution [211].
	Multivitamin solution containing vitamin D3 [138,193,212].
	Vitamin D3 [20,89,188,190,191,192,198,208,213].
	Stabilized aqueous vitamin D3 solution [197].
	Seven (22%) of the Australian units used vitamin D3, 90% of the units in New Zealand used multivitamin solution [199].
	2.9% vitamin D2; 52.2% vitamin D3; 33% multivitamin solution [9].
	Multivitamin solution containing vitamin D2, A, E, and C or vitamin D2 solution [182].
	Unspecified type of vitamin D used [8,19,41,51,71,97,98,180,194,196,200,211].
Route of administration	Orally, using a 1 mL syringe [211].
	Orally, using a brown 1 mL syringe [191].
	Orally, drops [20,89,180,182,193,197,198,212].
	Orally [71,97,98,138,196].
	Orogastric tube [8,19].
	Orogastric tube or orally [41,190,213].
	Orogastric tube, before feeding [51,189].
	Orogastric tube or orally, mixed with feedings [192].
	In human milk fortifier or formula [184].
	Enteral, mixed with feeding, and divided at 6 h [208].
	Not specified [21,188,194,196,199,200,204].
Vitamin D target for supplementation	>75 nmol/L [191].
	>50 nmol/L [21].
	A minimum of 50 nmol/L up to a maximum of 120 nmol/L [182].
	≥24 ng/mL [81].
	>30 ng/mL [85,189,192].
	30–60 ng/mL [198].
	Not specified [8,9,19,20,41,71,97,98,138,180,185,189,194,198,200,201,205,209].

**Table 3:** Recommendations for vitamin D supplementation in preterm infants.

Recommendations for Vitamin D Supplementation in Preterm Infants by Medical Societies	Vitamin D IU/Day
IOM + WHO [4]	400–1000
AAP [9]	200–400 IU up to max 1000 * (200 IU/day from PN/feeds)
ESPGHAN (2023) [61]	800–1000
ESPGHAN (2023) [61]	400–700 IU/kg/day up to maximum 1000 UI/day
Poland [215]	Preterm infants < 32 weeks of gestation: 800 IU/day if enteral feeding is possible, from the first day of life, irrespective of the feeding method; vitamin D in the diet should be calculated from the second month of life; vitamin D supplementation should be monitored with the first evaluation at 4 weeks of age, with monitoring continued after discharge; doses over 1000 IU/day may pose the risk of overdose, particularly in preterm infants with birth weight < 1000 g. Preterm infants with GA of 33–36 weeks: 400 IU/day; no routine evaluation is necessary; vitamin D supplementation under control should be considered if risk factors for VDD are present (parenteral nutrition or ketoconazole or anticonvulsants treatment > 2 weeks; cholestasis; birth weight < 1500 g)

* If infants tolerate full enteral feeding and achieve a weight > 1500 g. Legend: PN—parenteral nutrition; Institute of Medicine (IOM) [4]; World Health Organization (WHO); American Association of Pediatrics (AAP) [9]; European Society for Pediatric Gastroenterology Hepatology and Nutrition (ESPGHAN) [61].

## Data Availability

Not applicable.

## Abbreviations

The following abbreviations are used in this manuscript:

MDPI	Multidisciplinary Digital Publishing Institute
DOAJ	Directory of open access journals
TLA	Three letter acronym
LD	Linear dichroism

## References

[1] Brustad N., Yousef S., Stokholm J., Bønnelykke K., Bisgaard H., Chawes B.L. (2022). Safety of High-Dose Vitamin D Supplementation Among Children Aged 0 to 6 Years: A Systematic Review and Meta-analysis. JAMA Netw. Open.

[2] Sotunde O.F., Laliberte A., Weiler H.A. (2019). Maternal risk factors and newborn infant vitamin D status: A scoping literature review. Nutr. Res..

[3] Holick M.F., Binkley N.C., Bischoff-Ferrari H.A., Gordon C.M., Hanley D.A., Heaney R.P., Murad M.H., Weaver C.M., Endocrine Society (2011). Evaluation, treatment, and prevention of vitamin D deficiency: An Endocrine Society clinical practice guideline. J. Clin. Endocrinol. Metab..

[4] Bikle D.D. (2020). Vitamin D: Newer Concepts of Its Metabolism and Function at the Basic and Clinical Level. J. Endocr. Soc..

[5] Cui A., Zhang T., Xiao P., Fan Z., Wang H., Zhuang Y. (2023). Global and regional prevalence of vitamin D deficiency in population-based studies from 2000 to 2022: A pooled analysis of 7.9 million participants. Front. Nutr..

[6] Munns C.F., Shaw N., Kiely M., Specker B.L., Thacher T.D., Ozono K., Michigami T., Tiosano D., Mughal M.Z., Mäkitie O. (2016). Global Consensus Recommendations on Prevention and Management of Nutritional Rickets. J. Clin. Endocrinol. Metab..

[7] Stoffers A.J., Weber D.R., Levine M.A. (2022). An Update on Vitamin D Deficiency in the twenty-first century: Nature and nurture. Curr. Opin. Endocrinol. Diabetes Obes..

[8] Aristizabal N., Holder M.P., Durham L., Ashraf A.P., Taylor S., Salas A.A. (2023). Safety and Efficacy of Early Vitamin D Supplementation in Critically Ill Extremely Preterm Infants: An Ancillary Study of a Randomized Trial. J. Acad. Nutr. Diet..

[9] Romero-Lopez M., Naik M., Holzapfel L.F., Tyson J.E., Pedroza C., Ahmad K.A., Rysavy M.A., Carlo W.A., Zhang Y., Tibe C. (2024). Enteral nutritional practices in extremely preterm infants: A survey of U.S. NICUs. J. Perinatol..

[10] Kashaki M., Mohammadi Z., Mazouri A., Norouzi E. (2023). Comparison of the Effect of Oral Versus Parenteral Vitamin D on Serum Levels of Vitamin D in Premature Infants with Vitamin D Deficiency. J. Compr. Ped..

[11] Hollis B.W., Wagner C.L. (2022). Substantial Vitamin D Supplementation Is Required during the Prenatal Period to Improve Birth Outcomes. Nutrients.

[12] Agarwal S., Kovilam O., Agrawal D.K. (2018). Vitamin D and its impact on maternal-fetal outcomes in pregnancy: A critical review. Crit. Rev. Food Sci. Nutr..

[13] Palacios C., Kostiuk L.L., Cuthbert A., Weeks J. (2024). Vitamin D supplementation for women during pregnancy. Cochrane Database Syst. Rev..

[14] Treiber M., Mujezinović F., PečovnikBalon B., Gorenjak M., Maver U., Dovnik A. (2020). Association between umbilical cord vitamin D levels and adverse neonatal outcomes. J. Int. Med. Res..

[15] Lian R.H., Qi P.A., Yuan T., Yan P.J., Qiu W.W., Wei Y., Hu Y.G., Yang K.H., Yi B. (2021). Systematic review and meta-analysis of vitamin D deficiency in different pregnancy on preterm birth: Deficiency in middle pregnancy might be at risk. Medicine.

[16] Qin L.L., Lu F.G., Yang S.H., Xu H.L., Luo B.A. (2016). Does Maternal Vitamin D Deficiency Increase the Risk of Preterm Birth: A Meta-Analysis of Observational Studies. Nutrients.

[17] Matejek T., Zemankova J., Malakova J., Cermakova E., Skalova S., Palicka V. (2022). Severe vitamin D deficiency in preterm infants: Possibly no association with clinical outcomes?. Matern. Fetal Neonatal Med..

[18] Yang Y., Li Z., Yan G., Jie Q., Rui C. (2018). Effect of different doses of vitamin D supplementation on preterm infants—An updated meta-analysis. J. Matern. Fetal Neonatal Med..

[19] Tofe-Valera I., Pérez-Navero J.L., Caballero-Villarraso J., Cañete M.D., Villa-Jiménez R., De la Torre-Aguilar M.J. (2023). Vitamin d deficiency with high parathyroid hormone levels is related to late onset SEPSIS among preterm infants. BMC Pregnancy Childbirth.

[20] Matejek T., Navratilova M., Zaloudkova L., Malakova J., Maly J., Skalova S., Palicka V. (2020). Vitamin D status of very low birth weight infants at birth and the effects of generally recommended supplementation on their vitamin D levels at discharge. J. Matern. Fetal Neonatal Med..

[21] Zung A., Topf-Olivestone C., Shinwell E.S., Hofi L., Juster-Reicher A., Flidel-Rimon O. (2020). Reassessing vitamin D supplementation in preterm infants: A prospective study and review of the literature. J. Pediatr. Endocrinol. Metab..

[22] Dhandai R., Jajoo M., Singh A., Mandal A., Jain R. (2018). Association of vitamin D deficiency with an increased risk of late-onset neonatal sepsis. Paediatr. Int. Child Health.

[23] Abrams S.A. (2020). Vitamin D in Preterm and Full-Term Infants. Ann. Nutr. Metab..

[24] Budhwar S., Verma P., Verma R., Gupta S., Rai S., Rajender S., Singh K. (2020). Altered cord serum 25-hydroxyvitamin D signaling and placental inflammation is associated with pre-term birth. Am. J. Reprod. Immunol..

[25] Yi X., Liu J., Li Y., He H., Zhu X. (2023). Neonatal 25-hydroxy vitamin D levels after birth and 2 to 4 weeks after vitamin D supplementation and their impacts on complications. Chin. J. Perinat. Med..

[26] Park H.W., Lim G., Park Y.M., Chang M., Son J.S., Lee R. (2020). Association between vitamin D level and bronchopulmonary dysplasia: A systematic review and meta-analysis. PLoS ONE.

[27] Zhao R., Zhou L., Wang S., Yin H., Yang X., Hao L. (2022). Effect of maternal vitamin D status on risk of adverse birth outcomes: A systematic review and dose-response meta-analysis of observational studies. Eur. J. Nutr..

[28] Kiely M.E., Wagner C.L., Roth D.E. (2020). Vitamin D in pregnancy: Where we are and where we should go. J. Steroid Biochem. Mol. Biol..

[29] Corcoy R., Mendoza L.C., Simmons D., Desoye G., Adelantado J.M., Chico A., Devlieger R., van Assche A., Galjaard S., Timmerman D. (2020). The DALI vitamin D randomized controlled trial for gestational diabetes mellitus prevention: No major benefit shown besides vitamin D sufficiency. Clin. Nutr..

[30] Liu Y., Ding C., Xu R., Wang K., Zhang D., Pang W., Tu W., Chen Y. (2022). Effects of vitamin D supplementation during pregnancy on offspring health at birth: A meta-analysis of randomized controlled trails. Clin. Nutr..

[31] Gallo S., McDermid J.M., Al-Nimr R.I., Hakeem R., Moreschi J.M., Pari-Keener M., Stahnke B., Papoutsakis C., Handu D., Cheng F.W. (2020). Vitamin D Supplementation during Pregnancy: An Evidence Analysis Center Systematic Review and Meta-Analysis. J. Acad. Nutr. Diet..

[32] (2020). WHO Antenatal Care Recommendations for a Positive Pregnancy Experience: Nutritional Interventions Update: Vitamin D Supplements During Pregnancy.

[33] Demay M.B., Pittas A.G., Bikle D.D., Diab D.L., Kiely M.E., Lazaretti-Castro M., Lips P., Mitchell D.M., Murad M.H., Powers S. (2024). Vitamin D for the Prevention of Disease: An Endocrine Society Clinical Practice Guideline. J. Clin. Endocrinol. Metab..

[34] Tous M., Villalobos M., Iglesias-Vázquez L., Fernández-Barrés S., Arija V. (2020). Vitamin D status during pregnancy and offspring outcomes: A systematic review and meta-analysis of observational studies. Eur. J. Clin. Nutr..

[35] Kurmangali Z., Abdykalykova B., Kurmangali A., Zhantagulov D., Terzic M. (2024). The Influence of Vitamin D on Pregnancy and Outcomes: Current Knowledge and Future Perspectives. Gynecol. Obstet. Investig..

[36] Grant W.B., Wimalawansa S.J., Pludowski P., Cheng R.Z. (2025). Vitamin D: Evidence-Based Health Benefits and Recommendations for Population Guidelines. Nutrients.

[37] Gioxari A., Papandreou P., Daskalou E., Kaliora A.C., Skouroliakou M. (2024). Association of Serum Calcium Levels of Preterm Neonates at Birth with Calcium Intake from Foods and Supplements by Bedridden Women during Pregnancy. Healthcare.

[38] Phiri C.B., Davis C.R., Grahn M., Gannon B.M., Kokinos B.P., Crenshaw T.D., Tanumihardjo S.A. (2024). Vitamin D Maintains Growth and Bone Mineral Density against a Background of Severe Vitamin A Deficiency and Moderate Toxicity in a Swine Model. Nutrients.

[39] Giustina A., Bilezikian J.P., Adler R.A., Banfi G., Bikle D.D., Binkley N.C., Bollerslev J., Bouillon R., Brandi M.L., Casanueva F.F. (2024). Consensus Statement on Vitamin D Status Assessment and Supplementation: Whys, Whens, and Hows. Endocr. Rev..

[40] Çetinkaya M., Çekmez F., Erener-Ercan T., Buyukkale G., Demirhan A., Aydemir G., Aydin F.N. (2015). Maternal/neonatal vitamin D deficiency: A risk factor for bronchopulmonary dysplasia in preterms?. J. Perinatol..

[41] Zhang X., Luo K., He X., Chen P. (2021). Association of vitamin D status at birth with pulmonary disease morbidity in very preterm infants. Pediatr. Pulmonol..

[42] Boskabadi H., Zakerihamidi M., Mehrad-Majd H., Ghoflchi S. (2024). Evaluation of vitamin D in the diagnosis of infants with respiratory distress, the clinical value: A systematic review and meta-analysis. Paediatr. Respir. Rev..

[43] Salas A.A., Woodfin T., Phillips V., Peralta-Carcelen M., Carlo W.A., Ambalavanan N. (2018). Dose-Response Effects of Early Vitamin D Supplementation on Neurodevelopmental and Respiratory Outcomes of Extremely Preterm Infants at 2 Years of Age: A Randomized Trial. Neonatology..

[44] Yates N., Gunn A.J., Bennet L., Dhillon S.K., Davidson J.O. (2021). Preventing Brain Injury in the Preterm Infant-Current Controversies and Potential Therapies. Int. J. Mol. Sci..

[45] Ma S.S., Zhu D.M., Yin W.J., Hao J.H., Huang K., Tao F.B., Tao R.X., Zhu P. (2021). The role of neonatal vitamin D in the association of prenatal depression with toddlers ADHD symptoms: A birth cohort study. J. Affect. Disord..

[46] Cho H., Lee Y., Oh S., Heo J.S. (2025). Risk factors and outcomes of vitamin D deficiency in very preterm infants. Pediatr. Neonatol..

[47] Sava F., Treszl A., Hajdú J., Toldi G., Rigó J., Tulassay T., Vásárhelyi B. (2016). Plasma vitamin D levels at birth and immune status of preterm infants. Immunobiology.

[48] Katz T.A., Vliegenthart R.J.S., Aarnoudse-Moens C.S.H., Leemhuis A.G., Beuger S., Blok G.J., van Brakel M.J.M., van den Heuvel M.E.N., van Kempen A.A.M.W., Lutterman C. (2022). Severity of Bronchopulmonary Dysplasia and Neurodevelopmental Outcome at 2 and 5 Years Corrected Age. J. Pediatr..

[49] Cutuli S.L., Ferrando E.S., Cammarota F., Franchini E., Caroli A., Lombardi G., Tanzarella E.S., Grieco D.L., Antonelli M., De Pascale G. (2024). Update on vitamin D role in severe infections and sepsis. J. Anesth. Analg. Crit. Care.

[50] Hauta-Alus H.H., Rosendahl J., Holmlund-Suila E.M., Valkama S.M., Enlund-Cerullo M., Nurhonen M., Kajantie E., Mäkitie O., Andersson S. (2024). Low-grade inflammation from prenatal period to age 6-8 years in a Vitamin D trial. Pediatr. Res..

[51] Fort P., Salas A.A., Nicola T., Craig C.M., Carlo W.A., Ambalavanan N. (2016). A Comparison of 3 Vitamin D Dosing Regimens in Extremely Preterm Infants: A Randomized Controlled Trial. J. Pediatr..

[52] Reyes M.L., Vizcaya C., Le Roy C., Loureiro C., Brinkmann K., Arancibia M., Campos L., Iturriaga C., Pérez-Mateluna G., Rojas-McKenzie M. (2024). Weekly Vitamin D Supplementation to Prevent Acute Respiratory Infections in Young Children at Different Latitudes: A Randomized Controlled Trial. J. Pediatr..

[53] Wagner C.L., Hollis B.W. (2022). The extraordinary metabolism of vitamin D. eLife.

[54] Ashley B., Simner C., Manousopoulou A., Jenkinson C., Hey F., Frost J.M., Rezwan F.I., White C.H., Lofthouse E.M., Hyde E. (2022). Placental uptake and metabolism of 25(OH)vitamin D determine its activity within the fetoplacental unit. eLife.

[55] Moon R.J., Green H.D., D’Angelo S., Godfrey K.M., Davies J.H., Curtis E.M., Cooper C., Harvey N.C. (2023). The effect of pregnancy vitamin D supplementation on offspring bone mineral density in childhood: A systematic review and meta-analysis. Osteoporos. Int..

[56] Moon R.J., D’ Angelo S., Curtis E.M., Ward K.A., Crozier S.R., Schoenmakers I., Javaid M.K., Bishop N.J., Godfrey K.M., Cooper C. (2024). Pregnancy vitamin D supplementation and offspring bone mineral density in childhood follow-up of a randomized controlled trial. Am. J. Clin. Nutr..

[57] Brustad N., Garland J., Thorsen J., Sevelsted A., Krakauer M., Vinding R.K., Stokholm J., Bønnelykke K., Bisgaard H., Chawes B.L. (2020). Effect of High-Dose vs Standard-Dose Vitamin D Supplementation in Pregnancy on Bone Mineralization in Offspring Until Age 6 Years: A Prespecified Secondary Analysis of a Double-Blinded, Randomized Clinical Trial. JAMA Pediatr..

[58] Percival M.A., Anderson K.B., Pasco J.A., Hosking S.M., Williams L.J., Holloway-Kew K.L., Wark J.D., Hyde N.K. (2024). Gestational vitamin D and offspring fracture risk: Do associations persist into mid adolescence?. Eur. J. Clin. Nutr..

[59] Percival M.A., Pasco J.A., Hosking S.M., Williams L.J., Holloway-Kew K.L., Wark J.D., Hyde N.K. (2021). Maternal vitamin D and offspring fracture risk: The Vitamin D in Pregnancy study. Arch. Osteoporos..

[60] Taylor S.N. (2024). Vitamin D for very preterm infants-determining the how, when, and why. Pediatr. Res..

[61] Embleton N.D., Jennifer Moltu S., Lapillonne A., van den Akker C.H.P., Carnielli V., Fusch C., Gerasimidis K., van Goudoever J.B., Haiden N., Iacobelli S. (2023). Enteral Nutrition in Preterm Infants (2022): A Position Paper from the ESPGHAN Committee on Nutrition and Invited Experts. J. Pediatr. Gastroenterol. Nutr..

[62] Boddu S.K., Lankala R. (2022). Are we undertreating calcium deficiency in metabolic bone disease of prematurity? A case report and review. Front. Pediatr..

[63] Abrams S.A. (2021). Vitamin D and bone minerals in neonates. Early Hum. Dev..

[64] Sethi A., Priyadarshi M., Agarwal R. (2020). Mineral and bone physiology in the foetus, preterm and full-term neonates. Semin. Fetal Neonatal Med..

[65] Piersigilli F., Van Grambezen B., Hocq C., Danhaive O. (2020). Nutrients and Microbiota in Lung Diseases of Prematurity: The Placenta-Gut-Lung Triangle. Nutrients.

[66] Pinto M.R.C., Machado M.M.T., de Azevedo D.V., Correia L.L., Leite Á.J.M., Rocha H.A.L. (2022). Osteopenia of prematurity and associated nutritional factors: Case-control study. BMC Pediatr..

[67] Fernández L., Ruiz L., Jara J., Orgaz B., Rodríguez J.M. (2018). Strategies for the Preservation, Restoration and Modulation of the Human Milk Microbiota. Implications for Human Milk Banks and Neonatal Intensive Care Units. Front. Microbiol..

[68] Motte-Signoret E., Jlassi M., Lecoq L., Wachter P.Y., Durandy A., Boileau P. (2023). Early elevated alkaline phosphatase as a surrogate biomarker of ongoing metabolic bone disease of prematurity. Eur. J. Pediatr..

[69] Jiang H., Guo J., Li J., Li C., Du W., Canavese F., Baker C., Ying H., Hua J. (2023). Artificial Neural Network Modeling to Predict Neonatal Metabolic Bone Disease in the Prenatal and Postnatal Periods. JAMA Netw. Open..

[70] Chinoy A., Mughal M.Z., Padidela R. (2021). Metabolic bone disease of prematurity-National survey of current neonatal and paediatric endocrine approaches. Acta Paediatr..

[71] Avila-Alvarez A., Urisarri A., Fuentes-Carballal J., Mandiá N., Sucasas-Alonso A., Couce M.L. (2020). Metabolic Bone Disease of Prematurity: Risk Factors and Associated Short-Term Outcomes. Nutrients.

[72] Pharande P., Pammi M., Collins C.T., Zhou S.J., Abrams S.A. (2015). Vitamin D supplementation for prevention of vitamin D deficiency in preterm and low birth weight infants. Cochrane Database Syst. Rev..

[73] Sweet D.G., Carnielli V.P., Greisen G., Hallman M., Klebermass-Schrehof K., Ozek E., Te Pas A., Plavka R., Roehr C.C., Saugstad O.D. (2023). European Consensus Guidelines on the Management of Respiratory Distress Syndrome: 2022 Update. Neonatology.

[74] Desai R.K., Yildiz Atar H., Lakshminrusimha S., Ryan R.M. (2024). Use of surfactant beyond respiratory distress syndrome, what is the evidence?. J. Perinatol..

[75] Fandiño J., Toba L., González-Matías L.C., Diz-Chaves Y., Mallo F. (2019). Perinatal Undernutrition, Metabolic Hormones, and Lung Development. Nutrients.

[76] Lykkedegn S., Sorensen G.L., Beck-Nielsen S.S., Christesen H.T. (2015). The impact of vitamin D on fetal and neonatal lung maturation. A systematic review. Am. J. Physiol. Lung Cell Mol. Physiol..

[77] Lykkedegn S., Sorensen G.L., Beck-Nielsen S.S., Pilecki B., Duelund L., Marcussen N., Christesen H.T. (2016). Vitamin D Depletion in Pregnancy Decreases Survival Time, Oxygen Saturation, Lung Weight and Body Weight in Preterm Rat Offspring. PLoS ONE.

[78] Sakurai R., Singh H., Wang Y., Harb A., Gornes C., Liu J., Rehan V.K. (2021). Effect of Perinatal Vitamin D Deficiency on Lung Mesenchymal Stem Cell Differentiation and Injury Repair Potential. Am. J. Respir. Cell Mol. Biol..

[79] Chen C., Weng H., Zhang X., Wang S., Lu C., Jin H., Chen S., Liu Y., Sheng A., Sun Y. (2020). Low-Dose Vitamin D Protects Hyperoxia-Induced Bronchopulmonary Dysplasia by Inhibiting Neutrophil Extracellular Traps. Front. Pediatr..

[80] Mandell E.W., Ryan S., Seedorf G.J., Gonzalez T., Smith B.J., Fleet J.C., Abman S.H. (2020). Maternal Vitamin D Deficiency Causes Sustained Impairment of Lung Structure and Function and Increases Susceptibility to Hyperoxia-induced Lung Injury in Infant Rats. Am. J. Respir. Cell Mol. Biol..

[81] Liu W., Xu P. (2023). The association of serum vitamin D level and neonatal respiratory distress syndrome. Ital. J. Pediatr..

[82] Boskabadi H., Mamoori G., Khatami S.F., Faramarzi R. (2018). Serum level of vitamin D in preterm infants and its association with premature-related respiratory complications: A case-control study. Electron. Physician..

[83] Mohamed Hegazy A., Mohamed Shinkar D., Refaat Mohamed N., Abdalla Gaber H. (2018). Association between serum 25 (OH) vitamin D level at birth and respiratory morbidities among preterm neonates. Matern. Fetal Neonatal Med..

[84] Al-Beltagi M., Rowiesha M., Elmashad A., Elrifaey S.M., Elhorany H., Koura H.G. (2020). Vitamin D status in preterm neonates and the effects of its supplementation on respiratory distress syndrome. Pediatr. Pulmonol..

[85] Kim Y.J., Lim G., Lee R., Chung S., Son J.S., Park H.W. (2023). Association between vitamin D level and respiratory distress syndrome: A systematic review and meta-analysis. PLoS ONE.

[86] GhehsarehArdastani A., Hashemi E., Beheshtinejad M., Dorostkar R. (2020). Comparison of 25- Hydroxy Vitamin D Levels in Premature Infants with and without Respiratory Distress. Iran. J. Neonatol..

[87] Dogan P., Ozkan H., Koksal N., Bagci O., Varal I.G. (2020). Vitamin D deficiency and its effect on respiratory distress syndrome in premature infants: Results from a prospective study in a tertiary care centre. Afr. Health Sci..

[88] Yu R.Q., Chen D.Z., Hao X.Q., Jiang S.H., Fang G.D., Zhou Q. (2017). Relationship between serum 25(OH)D levels at birth and respiratory distress syndrome in preterm infants. Zhongguo Dang Dai Er Ke Za Zhi.

[89] Kim I., Kim S.S., Song J.I., Yoon S.H., Park G.Y., Lee Y.W. (2019). Association between vitamin D level at birth and respiratory morbidities in very-low-birth-weight infants. Korean J. Pediatr..

[90] Baldan E., Yarcı E. (2022). Serum 25-Hydroxyvitamin D Levels in Preterm Infants Born at Gestational Age of ≤32 Weeks and Prematurity-related Morbidities and Complications. J. Behcet Uz Child. S Hosp..

[91] Al-Matary A., AlMalki Y., Khalil S., AlHulaimi E. (2021). The potential effects of vitamin D deficiency on respiratory distress syndrome among preterm infants. Clin. Nutr. Espen..

[92] Yangin Ergon E., Dorum B.A., Balki H.G., Bako D., Alkan Ozdemir S. (2024). A Prospective Cross-Sectional Study on the Vitamin D Status of Neonates and the Impact of Neonates’ Standard Vitamin D Supplementation on Neonatal Morbidities. Children.

[93] Wang H., Du Y., Wu Z., Geng H., Zhu X., Zhu X. (2022). Serum Vitamin D Insufficiency in Hospitalized Full-Term Neonates at a Tertiary Hospital in Eastern China. Front. Pediatr..

[94] Laborie S., Bonjour M., Bacchetta J., Mauras M., Butin M. (2023). Is 25OH Vitamin D Excess before 36 Weeks Corrected Age an Independent Risk Factor for Bronchopulmonary Dysplasia or Death?. Nutrients.

[95] Lu T., Liang B., Jia Y., Cai J., Wang D., Liu M., He B., Wang Q. (2021). Relationship between bronchopulmonary dysplasia, long-term lung function, and vitamin D level at birth in preterm infants. Transl. Pediatr..

[96] Wang S., Wang M., Yu X., Cao C., Ding Y., Lv M., Liu Y., Chu M., Fang K., Liao Z. (2024). Nonlinear relationship between vitamin D status on admission and bronchopulmonary dysplasia in preterm infants. Pediatr. Res..

[97] Yu H., Fu J., Feng Y. (2022). Utility of umbilical cord blood 25-hydroxyvitamin D levels for predicting bronchopulmonary dysplasia in preterm infants with very low and extremely low birth weight. Front. Pediatr..

[98] Ge H., Qiao Y., Ge J., Li J., Hu K., Chen X., Cao X., Xu X., Wang W. (2022). Effects of early vitamin D supplementation on the prevention of bronchopulmonary dysplasia in preterm infants. Pediatr. Pulmonol..

[99] Cui X., Fu J. (2023). Early prediction of bronchopulmonary dysplasia: Can noninvasive monitoring methods be essential?. ERJ Open Res..

[100] Singh M., Alsaleem M., Gray C.P. (2018). Neonatal Sepsis.

[101] Kariniotaki C., Thomou C., Gkentzi D., Panteris E., Dimitriou G., Hatzidaki E. (2024). Neonatal Sepsis: A Comprehensive Review. Antibiotics.

[102] Gallo D., Baci D., Kustrimovic N., Lanzo N., Patera B., Tanda M.L., Piantanida E., Mortara L. (2023). How Does Vitamin D Affect Immune Cells Crosstalk in Autoimmune Diseases?. Int. J. Mol. Sci..

[103] Bouillon R., Marcocci C., Carmeliet G., Bikle D., White J.H., Dawson-Hughes B., Lips P., Munns C.F., Lazaretti-Castro M., Giustina A. (2019). Skeletal and Extraskeletal Actions of Vitamin D: Current Evidence and Outstanding Questions. Endocr. Rev..

[104] White J.H. (2022). Emerging Roles of Vitamin D-Induced Antimicrobial Peptides in Antiviral Innate Immunity. Nutrients.

[105] Martens P.J., Gysemans C., Verstuyf A., Mathieu A.C. (2020). Vitamin D’s Effect on Immune Function. Nutrients.

[106] Wimalawansa S.J. (2023). Physiological Basis for Using Vitamin D to Improve Health. Biomedicines.

[107] Wimalawansa S.J. (2024). Physiology of Vitamin D-Focusing on Disease Prevention. Nutrients.

[108] McNally J.D., Iliriani K., Pojsupap S., Sampson M., O’hearn K., McIntyre L., Fergusson D., Menon K. (2015). Rapid normalization of vitamin D levels: A meta-analysis. Pediatrics.

[109] De Pascale G., Vallecoccia M.S., Schiattarella A., Di Gravio V., Cutuli S.L., Bello G., Montini L., Pennisi M.A., Spanu T., Zuppi C. (2016). Clinical and microbiological outcome in septic patients with extremely low 25-hydroxyvitamin D levels at initiation of critical care. Clin. Microbiol. Infect..

[110] Zhou W., Mao S., Wu L., Yu J. (2018). Association Between Vitamin D Status and Sepsis. Clin. Lab..

[111] Li Y., Ding S. (2020). Serum 25-Hydroxyvitamin D and the risk of mortality in adult patients with Sepsis: A meta-analysis. BMC Infect. Dis..

[112] Haghdoost S., Pazandeh F., Darvish S., Khabazkhoob M., Huss R., Lak T.B. (2019). Association of serum vitamin D levels and urinary tract infection in pregnant women: A case control study. Eur. J. Obstet. Gynecol. Reprod. Biol..

[113] Yu W., Ying Q., Zhu W., Huang L., Hou Q. (2021). Vitamin D status was associated with sepsis in critically ill children: A PRISMA compliant systematic review and meta-analysis. Medicine.

[114] Durá-Travé T., Gallinas-Victoriano F. (2024). COVID-19 in Children and Vitamin D. Int. J. Mol. Sci..

[115] Jolliffe D.A., Camargo C.A., Sluyter J.D., Aglipay M., Aloia J.F., Bergman P., Bischoff-Ferrari H.A., Borzutzky A., Bubes V.Y., Damsgaard C.T. (2025). Vitamin D supplementation to prevent acute respiratory infections: Systematic review and meta-analysis of stratified aggregate data. Lancet Diabetes Endocrinol..

[116] Gan Y., You S., Ying J., Mu D. (2023). The Association between Serum Vitamin D Levels and Urinary Tract Infection Risk in Children: A Systematic Review and Meta-Analysis. Nutrients.

[117] Marino R., Misra M. (2019). Extra-Skeletal Effects of Vitamin D. Nutrients.

[118] Gao N., Raduka A., Rezaee F. (2023). Vitamin D3 protects against respiratory syncytial virus-induced barrier dysfunction in airway epithelial cells via PKA signaling pathway. Eur. J. Cell Biol..

[119] Cetinkaya M., Cekmez F., Buyukkale G., Erener-Ercan T., Demir F., Tunc T., Aydın F.N., Aydemir G. (2015). Lower vitamin D levels are associated with increased risk of early-onset neonatal sepsis in term infants. J. Perinatol..

[120] Kanth S.U., Reddy K.A., Srinivas G.A. (2016). Association between vitamin D levels and early onset sepsis in infants: A prospective observational study. Int. J. Contemp. Pediatr..

[121] Hagag A.A., El Frargy M.S., Houdeeb H.A. (2020). Therapeutic Value of Vitamin D as an Adjuvant Therapy in Neonates with Sepsis. Infect. Disord. Drug Targets..

[122] Gamal T.S., Madiha A.S., Hanan M.K., Abdel-Azeem M.E., Marian G.S. (2017). Neonatal and Maternal 25-OH Vitamin D Serum Levels in Neonates with Early-Onset Sepsis. Children.

[123] Czech-Kowalska J. (2020). Mineral and nutritional requirements of preterm infant. Semin. Fetal Neonatal Med..

[124] Kamsiah K., Hasibuan B.S., Arto K.S. (2021). The Relationship between Vitamin D Levels and Clinical Outcomes of Neonatal Sepsis in Haji Adam Malik Hospital Medan, Indonesia. Open Access Maced. J. Med. Sci..

[125] Abdel-Hady H., Yahia S., Megahed A., Mosbah A., Seif B., Nageh E., Bhattacharjee I., Aly H. (2019). Mediators in Preterm Infants with Late-onset Sepsis: A Randomized Controlled Trial. J. Pediatr. Gastroenterol. Nutr..

[126] Dammann O., Hartnett M.E., Stahl A. (2023). Retinopathy of prematurity. Dev. Med. Child. Neurol..

[127] Yin X., Xu S., Zhang X., Li L., Xi H., Ma L., Sun M., Yang P., Li X., Jiang H. (2024). The association between serum 25-hydroxyvitamin D levels and retinopathy of prematurity in preterm infants. Front. Pediatr..

[128] Kabataş E.U., Dinlen N.F., Zenciroğlu A., Dilli D., Beken S., Okumuş N. (2017). Relationship between serum 25-hydroxy vitamin D levels and retinopathy of prematurity. Scott. Med. J..

[129] Gorimanipalli B., Shetty R., Sethu S., Khamar P. (2023). Vitamin D and eye: Current evidence and practice guidelines. Indian. J. Ophthalmol..

[130] Jamali N., Wang S., Darjatmoko S.R., Sorenson C.M., Sheibani N. (2017). Vitamin D receptor expression is essential during retinal vascular development and attenuation of neovascularization by 1, 25(OH)2D3. PLoS ONE.

[131] Jamali N., Song Y.S., Sorenson C.M., Sheibani N. (2019). 1,25(OH)2D3 regulates the proangiogenic activity of pericyte through VDR-mediated modulation of VEGF production and signaling of VEGF and PDGF receptors. FASEB Bioadv..

[132] Song Y.S., Jamali N., Sorenson C.M., Sheibani N. (2023). Vitamin D Receptor Expression Limits the Angiogenic and Inflammatory Properties of Retinal Endothelial Cells. Cells.

[133] Murugeswari P., Vinekar A., Prakalapakorn S.G., Anandula V.R., Subramani M., Vaidya T.A., Nair A.P., Jayadev C., Ghosh A., Kumaramanickavel G. (2023). Correlation between tear levels of vascular endothelial growth factor and vitamin D at retinopathy of prematurity stages in preterm infants. Sci. Rep..

[134] Deb D., Annamalai R., Muthayya M. (2022). Correlation of vitamin D levels with low gestational age and low birth weight in babies developing retinopathy of prematurity. Indian. J. Public Health.

[135] Boskabadi H., Abrishami M., Shoeibi N., Sanei Z., Moradi A., Zakerihamidi M. (2022). Comparison of Vitamin D Levels in Premature Infants with and without Retinopathy of Prematurity. Arch. Iran. Med..

[136] Lee C., Lau E., Chusilp S., Filler R., Li B., Zhu H., Yamoto M., Pierro A. (2019). Protective effects of vitamin D against injury in intestinal epithelium. Pediatr. Surg. Int..

[137] Meng D., Zhu W., Shi H.N., Lu L., Wijendran V., Xu W., Walker W.A. (2015). Toll-like receptor-4 in human and mouse colonic epithelium is developmentally regulated: A possible role in necrotizing enterocolitis. Pediatr. Res..

[138] Adnan M., Wu S.Y., Khilfeh M., Davis V. (2022). Vitamin D status in very low birth weight infants and response to vitamin D intake during their NICU stays: A prospective cohort study. J. Perinatol..

[139] Barut D., Akisu M., Koroglu O.A., Terek D., Ergin F., Onay H., Yalaz M., Kultursay N. (2023). The role of vitamin D receptor gene polymorphism in the development of necrotizing enterocolitis. Pediatr. Res..

[140] Wu Z., Liu D., Deng F. (2022). The Role of Vitamin D in Immune System and Inflammatory Bowel Disease. J. Inflamm. Res..

[141] Yang L., Fang Y., Zheng J., Zhu Q., Tang L., Xiong F. (2024). Correlation between serum vitamin D level and acute invasive enteritis in children. Immun. Inflamm. Dis..

[142] Cetinkaya M., Erener-Ercan T., Kalayci-Oral T., Babayiğit A., Cebeci B., Semerci S.Y., Buyukkale G. (2017). Maternal/neonatal vitamin D deficiency: A new risk factor for necrotizing enterocolitis in preterm infants?. J. Perinatol..

[143] Shi Y., Liu T., Zhao X., Yao L., Hou A., Fu J., Xue X. (2018). Vitamin D ameliorates neonatal necrotizing enterocolitis via suppressing TLR4 in a murine model. Pediatr. Res..

[144] Yang L.R., Li H., Zhang T., Zhao R.C. (2018). Relationship between vitamin D deficiency and necrotizing enterocolitis in preterm infants. Zhongguo Dang Dai Er Ke Za Zhi.

[145] Grant W.B., Karras S.N., Bischoff-Ferrari H.A., Annweiler C., Boucher B.J., Juzeniene A., Garland C.F., Holick M.F. (2016). Do studies reporting ’U’-shaped serum 25-hydroxyvitamin D-health outcome relationships reflect adverse effects?. Derm. Endocrinol..

[146] von Websky K., Hasan A.A., Reichetzeder C., Tsuprykov O., Hocher B. (2018). Impact of vitamin D on pregnancy-related disorders and on offspring outcome. J. Steroid Biochem. Mol. Biol..

[147] Morales E., Julvez J., Torrent M., Ballester F., Rodríguez-Bernal C.L., Andiarena A., Vegas O., Castilla A.M., Rodriguez-Dehli C., Tardón A. (2015). Vitamin D in Pregnancy and Attention Deficit Hyperactivity Disorder-like Symptoms in Childhood. Epidemiology.

[148] Sucksdorff M., Brown A.S., Chudal R., Surcel H.M., Hinkka-Yli-Salomäki S., Cheslack-Postava K., Gyllenberg D., Sourander A. (2021). Maternal Vitamin D Levels and the Risk of Offspring Attention-Deficit/Hyperactivity Disorder. J. Am. Acad. Child. Adolesc. Psychiatry.

[149] García-Serna A.M., Morales E. (2020). Neurodevelopmental effects of prenatal vitamin D in humans: Systematic review and meta-analysis. Mol. Psychiatry.

[150] Lee B.K., Eyles D.W., Magnusson C., Newschaffer C.J., McGrath J.J., Kvaskoff D., Ko P., Dalman C., Karlsson H., Gardner R.M. (2021). Developmental vitamin D and autism spectrum disorders: Findings from the Stockholm Youth Cohort. Mol. Psychiatry.

[151] Sass L., Vinding R.K., Stokholm J., Bjarnadóttir E., Noergaard S., Thorsen J., Sunde R.B., McGrath J., Bønnelykke K., Chawes B. (2020). High-Dose Vitamin D Supplementation in Pregnancy and Neurodevelopment in Childhood: A Prespecified Secondary Analysis of a Randomized Clinical Trial. JAMA Netw. Open..

[152] Specht I.O., Janbek J., Thorsteinsdottir F., Frederiksen P., Heitmann B.L. (2020). Neonatal vitamin D levels and cognitive ability in young adulthood. Eur. J. Nutr..

[153] Specht I.O., Thorsteinsdottir F., Walker K.C., Olsen J., Heitmann B.L. (2020). Neonatal vitamin D status and risk of childhood epilepsy. Epilepsia.

[154] Sammallahti S., Holmlund-Suila E., Zou R., Valkama S., Rosendahl J., Enlund-Cerullo M., Hauta-Alus H., Lahti-Pulkkinen M., El Marroun H., Tiemeier H. (2023). Prenatal maternal and cord blood vitamin D concentrations and negative affectivity in infancy. Eur. Child. Adolesc. Psychiatry.

[155] Sandboge S., Räikkönen K., Lahti-Pulkkinen M., Hauta-Alus H., Holmlund-Suila E., Girchenko P., Kajantie E., Mäkitie O., Andersson S., Heinonen K. (2023). Effect of Vitamin D3 Supplementation in the First 2 Years of Life on Psychiatric Symptoms at Ages 6 to 8 Years: A Randomized Clinical Trial. JAMA Netw. Open.

[156] Tuovinen S., Räikkönen K., Holmlund-Suila E., Hauta-Alus H., Helve O., Rosendahl J., Enlund-Cerullo M., Kajantie E., Valkama S., Viljakainen H. (2021). Effect of High-Dose vs Standard-Dose Vitamin D Supplementation on Neurodevelopment of Healthy Term Infants: A Randomized Clinical Trial. JAMA Netw. Open.

[157] Hou Q.Y., Lin M.Y., Yuan T.M. (2022). Vitamin D level in umbilical cord blood of late preterm infants and the effect of vitamin D3 supplementation on the behavioral development of infants and young children: A prospective randomized controlled study. Zhongguo Dang Dai Er Ke Za Zhi.

[158] Guo H., Xie J., Yu X., Tian Y., Guan M., Wei J. (2024). Effects of vitamin D supplementation on serum 25(OH)D3 levels and neurobehavioral development in premature infants after birth. Sci. Rep..

[159] Cameron L.K., Ledwaba-Chapman L., Voong K., Hampson G., Forni L.G., Seylanova N., Harrington D.J., Lim R., Bociek A., Yanzhong W. (2024). Vitamin D metabolism in critically ill patients with acute kidney injury: A prospective observational study. Crit. Care.

[160] Kumar J., McDermott K., Abraham A.G., Friedman L.A., Johnson V.L., Kaskel F.J., Furth S.L., Warady B.A., Portale A.A., Melamed M.L. (2016). Prevalence and correlates of 25-hydroxyvitamin D deficiency in the Chronic Kidney Disease in Children (CKiD) cohort. Pediatr. Nephrol..

[161] Sürmeli Döven S., Erdoğan S. (2021). Vitamin D deficiency as a risk factor for renal scarring in recurrent urinary tract infections. Pediatr. Int..

[162] Chesney R.W. (2016). Interactions of vitamin D and the proximal tubule. Pediatr. Nephrol..

[163] Ersan S., Celik A., Tanrisev M., Kose I., Cavdar Z., Unlu M., Kocak A., Ural C., Yilmaz B., Kose T. (2017). Pretreatment with paricalcitol attenuates level and expression of matrix metalloproteinases in a rat model of renal ischemia-reperfusion injury. Clin. Nephrol..

[164] Lee Y.Q., Collins C.E., Gordon A., Rae K.M., Pringle K.G. (2018). The Relationship between Maternal Nutrition during Pregnancy and Offspring Kidney Structure and Function in Humans: A Systematic Review. Nutrients.

[165] Miliku K., Voortman T., Franco O.H., McGrath J.J., Eyles D.W., Burne T.H., Hofman A., Tiemeier H., Jaddoe V.W. (2016). Vitamin D status during fetal life and childhood kidney outcomes. Eur. J. Clin. Nutr..

[166] Biswas S.A., Rukunuzzaman M., Biswas R.K., Rahman S.M.H., Alam M.S. (2025). Serum Vitamin D Status in Infants with Cholestatic Jaundice. Mymensingh Med. J..

[167] Asemi Z., Karamali M., Esmaillzadeh A. (2015). Favorable effects of vitamin D supplementation on pregnancy outcomes in gestational diabetes: A double blind randomized controlled clinical trial. Horm. Metab. Res..

[168] Fernando M., Coster T.G., Ellery S.J., Guingand D., Lim S., Harrison C.L., Teede H.J., Naderpoor N., Mousa A. (2020). Relationships between Total, Free and Bioavailable Vitamin D and Vitamin D Binding Protein in Early Pregnancy with Neonatal Outcomes: A Retrospective Cohort Study. Nutrients.

[169] Al-Banna S.M., Riad A.N., Anes S.S. (2020). The effect of Fenofibrate and Antioxidant Vitamins [D, E and C] in Treatment of Uncomplicated Neonatal Hyperbilirubinemia. Ann. Neo. J..

[170] Bhat J.A., Sheikh S.A., Ara R. (2021). Correlation of 25-hydroxy vitamin D level with neonatal hyperbilirubinemia in term healthy newborn: A prospective hospital-based observation study. Int. J. Pediatr. Adolesc. Med..

[171] Elfarargy M.S., Ali D.A., Al-Ashmawy G.M. (2019). Study of vitamin D and melatonin supplementation as adjuvant therapies in neonatal jaundice. Curr. Pediatr. Res..

[172] Aletayeb S.M., Dehdashtiyan M., Aminzadeh M., Malekyan A., Jafrasteh S. (2016). Comparison between maternal and neonatal serum vitamin D levels in term jaundiced and nonjaundiced cases. J. Chin. Med. Assoc..

[173] Mehrpisheh S., Memarian A., Mahyar A., Valiahdi N.S. (2018). Correlation between serum vitamin D level and neonatal indirect hyperbilirubinemia. BMC Pediatr..

[174] Huang J., Zhao Q., Li J., Meng J., Li S., Yan W., Wang J., Ren C. (2021). Correlation between neonatal hyperbilirubinemia and vitamin D levels: A meta-analysis. PLoS ONE.

[175] Zhou W., Wang P., Bai Y., Zhang Y., Shu J., Liu Y. (2023). Vitamin D metabolic pathway genes polymorphisms and vitamin D levels in association with neonatal hyperbilirubinemia in China: A single-center retrospective cohort study. BMC Pediatr..

[176] Afzal Z., Tayyab M., Rizwan W. (2024). Vitamin D Supplementation as Adjuvant with Phototherapy Is Beneficial in Neonatal Jaundice. Proceedings S.Z.M.C..

[177] Das G., Jain A., Gupta V., Shukla D. (2024). The Effect of Vitamin D Supplementation as Adjuvant to Phototherapy versus Phototherapy Alone on Neonatal Jaundice: A Randomised Controlled Trial. J. Clin. Diagn. Res..

[178] Mori J.D., Kassai M.S., Lebrão C.W., Affonso-Fonseca F.L., Sarni R.O.S., Suano-Souza F.I. (2023). Influence of umbilical cord vitamin D serum levels on the growth of preterm infants. Nutrition.

[179] Nabiel N., Hari Wahyu N., Annang Giri M. (2024). Vitamin D Deficiency is Associated with Hypocalcemia in Preterm Infants. Mol. Cell Biomed. Sci..

[180] Choudhury K.A., Kumar M., Tripathi S., Singh S.N., Singh K., Singh V.K. (2021). Vitamin D Status of Very Low Birth Weight Neonates at Baseline and Follow-up after Daily Intake of 800 IU Vitamin D. J. Trop. Pediatr..

[181] Jiang X., Lu J., Zhang Y., Teng H., Pei J., Zhang C., Guo B., Yin J. (2021). Association between maternal vitamin D status with pregnancy outcomes and offspring growth in a population of Wuxi, China. Asia Pac. J. Clin. Nutr..

[182] Mathilde M., Butin M., Pascal R., Plaisant F., Laborie S., Bacchetta J. (2022). Local protocol helped to deliver vitamin D levels more accurately in preterm infants. Acta Paediatr..

[183] Park S.H., Lee G.M., Moon J.E., Kim H.M. (2015). Severe vitamin D deficiency in preterm infants: Maternal and neonatal clinical features. Korean J. Pediatr..

[184] Kołodziejczyk-Nowotarska A., Bokiniec R., Seliga-Siwecka J. (2021). Monitored Supplementation of Vitamin D in Preterm Infants: A Randomized Controlled Trial. Nutrients.

[185] Yuet-ling Tung J., Calabria A.C., Kelly A. (2017). Vitamin D Status in Premature Infants at Risk of Metabolic Bone Disease of Prematurity. Matern. Pediatr. Nutr..

[186] Patel P., Pollock N., Bhatia J. (2017). Vitamin D and Bone Health in Very Low and Extremely Low Birth Weight Infants. J. Clin. Nutr. Metab..

[187] Kiely M., O’Donovan S.M., Kenny L.C., Hourihane J.O., Irvine A.D., Murray D.M. (2017). Vitamin D metabolite concentrations in umbilical cord blood serum and associations with clinical characteristics in a large prospective mother-infant cohort in Ireland. J. Steroid Biochem. Mol. Biol..

[188] Chen Y.W., Chang Y.J., Chen L.J., Lee C.H., Hsiao C.C., Chen J.Y., Chen H.N. (2024). Neurodevelopment Outcomes in Very-Low-Birth-Weight Infants with Metabolic Bone Disease at 2 Years of Age. Children.

[189] Cho S.Y., Park H.K., Lee H.J. (2017). Efficacy and safety of early supplementation with 800 IU of vitamin D in very preterm infants followed by underlying levels of vitamin D at birth. Ital. J. Pediatr..

[190] Paw D., Bokiniec R., Kołodziejczyk-Nowotarska A. (2024). High Initial Dose of Monitored Vitamin D Supplementation in Preterm Infants (HIDVID Trial): Study Protocol for a Randomized Controlled Study. Nutrients.

[191] Anderson-Berry A., Thoene M., Wagner J., Lyden E., Jones G., Kaufmann M., Van Ormer M., Hanson C. (2017). Randomized trial of two doses of vitamin D3 in preterm infants <32 weeks: Dose impact on achieving desired serum 25(OH)D3 in a NICU population. PLoS ONE.

[192] Bozkurt O., Uras N., Sari F.N., Atay F.Y., Sahin S., Alkan A.D., Canpolat F.E., Oguz S.S. (2017). Multi-dose vitamin d supplementation in stable very preterm infants: Prospective randomized trial response to three different vitamin D supplementation doses. Early Hum. Dev..

[193] Kazzi S.N.J., Karnati S., Puthuraya S., Thomas R. (2018). Vitamin D deficiency and respiratory morbidity among African American very low birth weight infants. Early Hum. Dev..

[194] Dursun M., Ozcabi B., Sariaydin M. (2022). Factors Affecting Metabolic Bone Disease of Prematurity: Is Hypothyroxinemia Included?. Sisli Etfal Hastan. Tip. Bul..

[195] Angelika D., Etika R., Mapindra M.P., Utomo M.T., Rahardjo P., Ugrasena I.D.G. (2021). Associated neonatal and maternal factors of osteopenia of prematurity in low resource setting: A cross-sectional study. Ann. Med. Surg..

[196] Kavurt S., Demirel N., Yücel H., Unal S., Yıldız Y.T., Bas A.Y. (2021). Evaluation of radiologic evidence of metabolic bone disease in very low birth weight infants at fourth week of life. J. Perinatol..

[197] Aly H., Mohsen L., Bhattacharjee I., Malash A., Atyia A., Elanwary S., El Hawary R. (2019). Vitamin D Supplementation and T Cell Regulation in Preterm Infants: A Randomized Controlled Trial. J. Pediatr. Gastroenterol. Nutr..

[198] Munshi U.K., Graziano P.D., Meunier K., Ludke J., Rios A. (2018). Serum 25 Hydroxy Vitamin D Levels in Very Low Birth Weight Infants Receiving Oral Vitamin D Supplementation. J. Pediatr. Gastroenterol. Nutr..

[199] Oliver C., Watson C., Crowley E., Gilroy M., Page D., Weber K., Messina D., Cormack B. (2019). Vitamin and Mineral Supplementation Practices in Preterm Infants: A Survey of Australian and New Zealand Neonatal Intensive and Special Care Units. Nutrients.

[200] Sabroske E.M., Payne D.H., Stine C.N., Kathen C.M., Sollohub H.M., Kohlleppel K.L., Lorbieski P.L., Carney J.E., Motta C.L., Pierce M.R. (2020). Effect on metabolic bone disease markers in the neonatal intensive care unit with implementation of a practice guideline. J. Perinatol..

[201] O’Connor D.L., Kiss A., Tomlinson C., Bando N., Bayliss A., Campbell D.M., Daneman A., Francis J., Kotsopoulos K., Shah P.S. (2018). Nutrient enrichment of human milk with human and bovine milk-based fortifiers for infants born weighing <1250 g: A randomized clinical trial. Am. J. Clin. Nutr..

[202] Chin L.K., Doan J., Teoh Y.S., Stewart A., Forrest P., Simm P.J. (2018). Outcomes of standardised approach to metabolic bone disease of prematurity. J. Paediatr. Child. Health.

[203] Faienza M.F., D’Amato E., Natale M.P., Grano M., Chiarito M., Brunetti G., D’Amato G. (2019). Metabolic Bone Disease of Prematurity: Diagnosis and Management. Front. Pediatr..

[204] Jung J.H., Kim E.A., Lee S.Y., Moon J.E., Lee E.J., Park S.H. (2021). Vitamin D Status and Factors Associated with Vitamin D Deficiency during the First Year of Life in Preterm Infants. Nutrients.

[205] Irvine J., Ward L.M. (2022). Preventing symptomatic vitamin D deficiency and rickets among Indigenous infants and children in Canada. Paediatr. Child. Health.

[206] Hekimoğlu B., Erin R., Yılmaz H.K. (2023). Comparison of cord blood and 6-month-old vitamin D levels of healthy term infants supplemented with 400 IU/day dose of vitamin D. Eur. J. Clin. Nutr..

[207] Lavassani E., Tauber K.A., Cerone J.B., Ludke J., Munshi U.K. (2024). Human milk-derived versus bovine milk-derived fortifier use in very low birth weight infants: Growth and vitamin D status. Front. Pediatr..

[208] Romero-Lopez M., Tyson J.E., Naik M., Pedroza C., Holzapfel L.F., Avritscher E., Mosquera R., Khan A., Rysavy M. (2024). Randomized controlled trial of enteral vitamin D supplementation (ViDES) in infants <28 weeks gestational age or <1000 g birth weight: Study protocol. Trials.

[209] Taylor S.N., Wahlquist A., Wagner C.L., Ramakrishnan V., Ebeling M., Hollis B.W. (2018). Functional indicators of vitamin D adequacy for very low birth weight infants. J. Perinatol..

[210] Marinov D.B., Hristova D.N. (2021). Supplementation with vitamin D—Current recommendations. J. IMAB.

[211] Mathur N.B., Saini A., Mishra T.K. (2016). Assessment of Adequacy of Supplementation of Vitamin D in Very Low Birth Weight Preterm Neonates: A Randomized Controlled Trial. J. Trop. Pediatr..

[212] Tergestina M., Rebekah G., Job V., Simon A., Thomas N. (2016). A randomized double-blind controlled trial comparing two regimens of vitamin D supplementation in preterm neonates. J. Perinatol..

[213] Tang W.Q., Ma N., Meng L.Y., Luo Y.W., Wang Y.J., Zhang D. (2023). Vitamin D supplementation improved physical growth and neurologic development of Preterm Infants receiving Nesting Care in the neonatal Intensive Care Unit. BMC Pediatr..

[214] Dima V., Iancu G., Bohiltea R.-E., Nastase l., Toma A.I., Varla V.-N., Davitoiu A.-M. (2022). Vitamin D supplementation—Still a subject of debate. Ro J. Med. Pract..

[215] Płudowski P., Kos-Kudła B., Walczak M., Fal A., Zozulińska-Ziółkiewicz D., Sieroszewski P., Peregud-Pogorzelski J., Lauterbach R., Targowski T., Lewiński A. (2023). Guidelines for Preventing and Treating Vitamin D Deficiency: A 2023 Update in Poland. Nutrients.

[216] Cirstoveanu C., Ionita I., Georgescu C., Heriseanu C., Vasile C.M., Bizubac M. (2024). Intermittent High-Dose Vitamin D3 Administration in Neonates with Multiple Comorbidities and Vitamin D Insufficiency. Children.

[217] Zozaya C., García-Serrano A., Fontecha J., Redondo-Bravo L., Sánchez-González V., Montes M.T., Saenz de Pipaón M. (2018). Fat Loss in Continuous Enteral Feeding of the Preterm Infant: How Much, What and When Is It Lost?. Nutrients.

[218] Abdelrahman K., Jarjour J., Hagan J., Yang H., Sutton D., Hair A. (2020). Optimizing Delivery of Breast Milk for Premature Infants: Comparison of Current Enteral Feeding Systems. Nutr. Clin. Pract..

[219] Pilz S., Trummer C., Theiler-Schwetz V., Grübler M.R., Verheyen N.D., Odler B., Karras S.N., Zittermann A., März W. (2022). Critical Appraisal of Large Vitamin D Randomized Controlled Trials. Nutrients.

[220] Gospodarska E., Ghosh Dastidar R., Carlberg C. (2023). Intervention Approaches in Studying the Response to Vitamin D3 Supplementation. Nutrients.

